# Cost-effective ^17^O enrichment and NMR spectroscopy of mixed-metal terephthalate metal–organic frameworks†
†Electronic supplementary information (ESI) available: Information on synthetic procedures, mass spectrometry, XRD, EDX and SEM experiments, DFT calculations, additional NMR spectra and characterisation of BDC samples and mixed-phase materials. See DOI: 10.1039/c7sc04649a


**DOI:** 10.1039/c7sc04649a

**Published:** 2017-11-23

**Authors:** Giulia P. M. Bignami, Zachary H. Davis, Daniel M. Dawson, Samuel A. Morris, Samantha E. Russell, David McKay, Richard E. Parke, Dinu Iuga, Russell E. Morris, Sharon E. Ashbrook

**Affiliations:** a School of Chemistry , EaStCHEM and Centre of Magnetic Resonance , University of St Andrews , North Haugh, St Andrews , Fife , KY16 9ST , UK . Email: sema@st-andrews.ac.uk ; Email: rem1@st-andrews.ac.uk; b UK 850 MHz Solid-State NMR Facility , Department of Physics , University of Warwick , Millburn House , Coventry , CV4 7AL , UK

## Abstract

^17^O solid-state NMR spectroscopy is employed to investigate the cation disorder in metal–organic frameworks containing two different types of metal cations. Although NMR offers exquisite sensitivity to the local, atomic-scale structure, making it an ideal tool for the characterisation of disordered materials, the low natural abundance of ^17^O (0.037%) necessitates expensive isotopic enrichment to acquire spectra on a reasonable timescale. Using dry gel conversion and a novel steaming method we show that cost-effective and atom-efficient enrichment of MOFs is possible, and that high-resolution ^17^O NMR spectra are sensitive both to the structural forms of the MOF and the presence of guest molecules. For mixed-metal forms of MIL-53, NMR can also provide information on the final composition of the materials (notably different to that of the initial starting material) and the preference for cation clustering/ordering within the MOFs. For Al, Ga MIL-53, the distribution of cations results in a mixed-pore form upon exposure to water, unlike the different structures seen for the corresponding end members. This work shows that as good levels of enrichment can be achieved at reasonable cost, ^17^O NMR spectroscopy should be an invaluable tool for the study of these important functional materials.

## Abstract 2

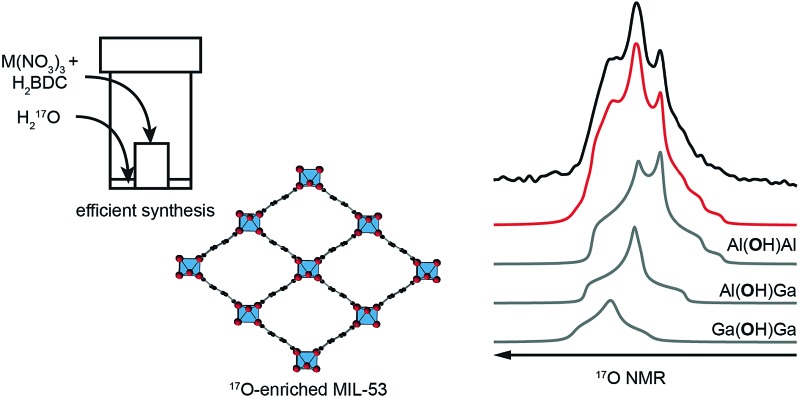
Cost-effective ^17^O enrichment of metal–organic frameworks enables the composition and disorder in mixed-metal materials to be determined using NMR spectroscopy.

## Introduction

**Fig. 1:**
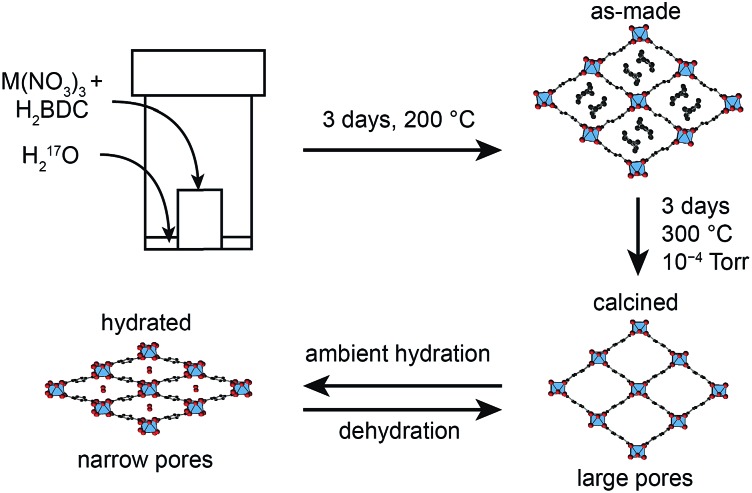
Scheme showing the breathing behaviour of Al MIL-53, starting from the as-made form synthesised using dry gel conversion (DGC), with disordered excess linker molecules in the pores. Upon calcination, the structure is in its empty open- (or large-)pore form and can undergo reversible hydration, resulting in a hydrated (closed- or narrow-pore) form. However, Ga MIL-53 remains in the closed-pore form upon dehydration.

Metal–organic frameworks (MOFs) are an important class of hybrid microporous materials, containing inorganic metal nodes and organic linkers. The MOF topology is governed by the coordination geometry of the nodes and the polytopic nature of the organic linkers, providing a variety of structures with high surface area and tunable pore sizes.^1^ Owing to these unique structural features, MOFs have been successfully employed in gas adsorption, storage and separation leading to applications in catalysis and drug delivery.^2^–^4^ Among the terephthalate-based MOFs, MIL-53 (MIL = Materiaux Insitut Lavoisier) is known for its reversible “breathing” behaviour, characterised by large variations in pore size (up to 40%).^5^,^6^ MIL-53 was originally synthesised as a Cr^3+^ terephthalate,^7^ but can also be prepared with Al,^8^ Fe,^9^ In,^10^ Ga^11^ and Sc.^12^ The presence of different metal nodes in MIL-53 is known to affect the exact structural forms observed.^9^,^11^–^16^ For example, for Al MIL-53, as shown in Fig. 1, the removal of excess linker molecules upon calcination of the as-made material leads to an empty large-pore form which can be reversibly interconverted to a narrow-pore hydrated form.

Mixed-metal MOFs containing more than one type of node are of great current interest.^17^–^20^ Mixed metals can be introduced either during synthesis or through post-synthetic transmetallation,^21^,^22^ and can significantly affect the properties of the materials produced. In MIL-53 the presence of mixed-metal centres has been shown to cause changes^17^–^19^ both in the structures adopted and in the reversibility of the breathing behaviour, suggesting further investigation is required to fully understand how the porous properties of MOFs can be controlled and tuned for target applications.

Cation disorder can pose a significant challenge for structural characterisation of a material, where commonly applied diffraction techniques often only provide information on the average structure. Techniques sensitive to local structure are preferable for studying this type of material. Solid-state nuclear magnetic resonance (NMR) spectroscopy^23^–^25^ has been widely applied to the study of MOFs,^26^–^28^ providing an element specific measurement that is sensitive to the local geometry and able to probe dynamics over a wide range of timescales. Studies have typically focussed on NMR of nuclei with spin quantum number *I* = 1/2, *i.e.*, ^1^H, ^13^C and ^15^N.^26^–^28^ This can provide information on the number, type and functionalization of the linkers present, and also on the presence, position and motion of many guest molecules. The quadrupolar (*i.e.*, *I* > 1/2) nature of many metal nodes provides additional challenges for NMR, but for diamagnetic species with sufficient receptivity and moderate quadrupolar broadening, such as ^11^B, ^27^Al and ^45^Sc, direct study of the metal coordination environment is also possible.^26^–^28^ Recent work has also demonstrated that wideline experiments^29^ can also be used to study more challenging nuclei in MOFs, such as ^69/71^Ga, ^91^Zr, ^47/49^Ti, ^115^In and ^139^La.^30^,^31^ However, for paramagnetic centres, including Fe^3+^, Cr^3+^ or Cu^2+^, study is typically restricted to the organic linker, although very rapid sample rotation is often required to reduce broadening arising from interactions with the unpaired electrons.^32^ In contrast, oxygen NMR has been relatively underexploited. This is perhaps surprising, given that oxygen atoms link the metal nodes and organic linkers in many MOFs, and are also present in the water and hydroxyl groups that are attached to, or even bridge, metal centres. Changes in pore structure (and the adsorption of molecules to free coordination sites at metal centres) might also be reflected in changes to the oxygen coordination geometry and, subsequently, in the NMR spectrum. The only NMR-active isotope, ^17^O, is quadrupolar (with *I* = 5/2), but has one of the smallest quadrupole moments known, typically reducing the quadrupolar broadening to such levels that, in principle, spectra can be acquired relatively easily, and with the added advantage that variation in the parameters that define the quadrupolar interaction (*i.e.*, magnitude, *C*_Q_, and asymmetry, *η*_Q_) provides additional structural information.^23^–^25^,^33^,^34^ However, spectral investigation is severely limited by the low natural abundance of ^17^O (0.037%). Therefore, for high-resolution spectroscopic investigation isotopic enrichment is vital, but the considerable cost of enriched reagents necessitates the development of synthetic procedures that are both cost- and atom-efficient and can be carried out at significantly reduced scale. Consequently, there has been relatively little ^17^O NMR of MOFs reported,^35^,^36^ despite its great potential. One possible approach to the cost-effective ^17^O enrichment of MOFs is dry gel conversion (DGC),^37^–^40^ due to the relatively small amount of solvent (μL) required compared to solvothermal synthesis (mL). This has been used for the synthesis of ^17^O-enriched Al MIL-53, as shown in Fig. 1,^36^ with Al(NO_3_)_3_ and terephthalic acid placed within a Teflon cup and sealed in an autoclave containing a small amount of H_2_^17^O.

Here, we show how DGC can be a cost- and atom-effective method to produce ^17^O-enriched mixed-metal MIL-53 materials. We then employ ^17^O NMR spectroscopy to probe cation disorder and understand the resulting effects on the breathing behaviour of the materials and the structural forms observed. We also demonstrate the limitations of the DGC approach, and investigate the use of an alternative enrichment procedure, involving post-synthesis steaming in ^17^O-enriched water vapour.^41^–^43^

## Experimental

### Synthetic procedures

MIL-53 materials were synthesised using DGC, as described in ref. 36, with the quantities of reagents given in the ESI.† The corresponding hydrated metal nitrates were used as the cation source and terephthalic acid (benzene-1,4-dicarboxylic acid, H_2_BDC) as the linker. The precursors were placed in a Teflon cup, which was placed inside a Teflon-lined autoclave containing ^17^O-enriched water (130 μL, 90% ^17^O, CortecNet). Upon sealing, the autoclave was placed in an oven and heated to 200 °C at autogenous pressure for 72 h. The resulting product was then filtered and washed with a minimum amount of water before leaving it overnight to dry in air. Compounds containing Sc^3+^ were yellow, and all other products where white. For Al, Ga and mixed Al, Ga syntheses this approach produced MIL-53 materials, but when Sc was included in any synthesis the smaller-pore MOF Sc_2_(BDC)_3_ was produced. Sc MIL-53 was synthesised *via* a solvothermal route, as outlined in ref. 12. For this, a solution of scandium nitrate hydrate (0.117 g), H_2_BDC (0.068 g), pyridine (0.487 g) and *N*,*N*-dimethylformamide (6 g) was stirred, transferred to a Teflon-lined autoclave and then heated to 190 °C at autogenous pressure for 40 h. The resulting compound was filtered and washed with ethanol before drying overnight at 60 °C. Sc MIL-53 was enriched *via* post-synthetic steaming, by placing the material into a Teflon-cup, which was placed inside a Teflon-lined autoclave containing 90% ^17^O-enriched water (130 μL) and heated to 200 °C at autogenous pressure for 72 h. The calcination of all samples (aside from Sc MIL-53) was performed by heating the compounds in a Buchi furnace at 300 °C under a dynamic vacuum of 10^–3^ to 10^–4^ Torr for 72 h, enabling the removal of unreacted linker and water from within the MOF pores. Dehydration was carried out in the same way but at a reduced temperature of 100 °C for 20 h. The calcined or dehydrated samples were then sealed in an ampule, under an argon atmosphere. Calcination of Sc MIL-53 was performed by heating the sample in a tube furnace, under N_2_ gas flow, at 350 °C for 12 h.

### Powder X-ray diffraction (XRD)

Powder XRD data were collected on a STOE STADIP instrument operated in capillary Debye–Scherrer mode equipped with a Cu X-ray tube, a primary beam monochromator (CuK_α1_) and a scintillation position-sensitive linear detector. Typically, 2*θ* ranges of 3–50° were investigated over 15 h of acquisition time and samples were packed in 0.7 or 0.5 mm glass capillaries. Capillaries containing calcined or dehydrated samples were sealed to avoid water adsorption during acquisition. All refinements were carried out using TOPAS v5.2. The Al/Ga ratio was determined by modelling both over a disordered site with a total occupancy of 1 and refining their individual occupancies with fixed isotropic displacement parameters. Each element was refined with a single isotropic displacement parameter to reduce over-parameterisation. The disordered organic found in the as-synthesised form was treated in the same fashion as in ref. 8; each carbon was treated with the same isotropic displacement parameter and occupancy factor.

### Nuclear magnetic resonance (NMR) spectroscopy

Solid-state NMR spectra were acquired using Bruker Avance III spectrometers equipped with either 14.1 or 20.0 T wide-bore magnets. Samples were packed into 3.2 mm ZrO_2_ rotors and magic angle spinning (MAS) spectra were acquired at spinning speeds of 12.5 kHz (^1^H, ^13^C), 16 kHz (^27^Al) and 20 kHz (^17^O) using a conventional 3.2 mm HX probe (for the spectra in Fig. S4.3b and S4.4b in the ESI,† the sample was packed into a 4 mm ZrO_2_ rotor and rotated at a spinning speed of 14 kHz using a 4 mm HX low-γ probe). Spectra were acquired at Larmor frequencies (at 14.1 T) of 150.87 MHz, 600.13 MHz and 156.34 MHz for ^13^C, ^1^H and ^27^Al, respectively, and for ^17^O at 81.34 MHz (14.1 T) and 115.3 MHz (20.0 T). Radiofrequency (rf) field strengths were calibrated to be 100 kHz (^1^H), 100 kHz (^27^Al) and 71 kHz (^17^O). Spectra are referenced to Si(CH_3_)_4_ for ^1^H and ^13^C (using a secondary reference of l-alanine (*δ*(**C**H_3_) = 20.5 ppm and *δ*(N**H**_3_) = 8.5 ppm)), Al(NO_3_)_3_ (aq) (0.5 M) for ^27^Al (using Al(acac)_3_ (*δ*_iso_ = 0 ppm) as a secondary reference) and H_2_O (^17^O) at room temperature. For ^13^C NMR experiments, transverse magnetisation was obtained by cross-polarisation (CP)^44^ from ^1^H using a 5 ms ramped contact pulse (ramped for ^1^H) and two-pulse phase modulation (TPPM)^45^^1^H decoupling (100 kHz) in acquisition. ^17^O and ^1^H MAS NMR 1D spectra were acquired using a rotor-synchronised spin echo^46^ pulse sequence to avoid baseline distortions. ^17^O double rotation (DOR)^47^,^48^ spectra were acquired at 20.0 T using a Samoson DOR probe. Experiments were carried out using a rf field strength of 50 kHz, with typical spinning speeds of 1250–1760 Hz for the outer rotor and 6300–8300 Hz for the inner rotor using, where possible, odd order sideband suppression.^49^,^50^ Isotropic centre bands were identified by recording a second spectrum at a different rotation rate. At 14.1 T, ^17^O MQMAS^51^ experiments were carried out using a triple-quantum z-filtered^52^ (0 → ±3 → 0 → –1) pulse sequence and are shown after a shearing transformation.^53^ At 20.0 T, experiments were carried out using a (0 → ±3 → ±1 → 0 → –1) sequence with double frequency sweeps^54^,^55^ employed for the ±3 → ±1 step (and are also shown after a shearing transformation). ^27^Al MQMAS spectra were acquired using a triple-quantum split-t_1_ shifted-echo pulse sequence.^53^ All spectra are referenced in the indirect dimension according to the convention described in ref. 56. The position of the centre of gravity of a resonance in the MQMAS experiment provides the mean isotropic chemical shift, *δ*_iso_, and the mean quadrupolar product, *P*_Q_ (where *P*_Q_ = *C*_Q_(1 + *η*_Q_^2^/3)^1/2^ and *C*_Q_ and *η*_Q_ are the quadrupolar coupling constant and asymmetry, respectively).^34^ Fitting the lineshapes extracted from the MQMAS spectra can also yield *δ*_iso_, *C*_Q_ and *η*_Q_.

### Scanning electron microscopy (SEM) and energy dispersive X-ray (EDX) spectroscopy

SEM and EDX measurements were carried out using a JSM-5600 conventional Scanning Electron Microscope with a tungsten filament electron source, equipped with a secondary electron detector for topographic contrast imaging and an Oxford Inca EDX system for compositional analysis. A working distance of 20 mm, a spot size of 40 and a voltage of 25 kV in the W filament were used. Samples were sieved prior to being placed on their sample holders to allow the dispersion and identification of isolated crystallites and then gold coated with a Quorum Q150R ES instrument to provide a conductive surface.

### Mass spectrometry

Samples were prepared by embedding the MOF powders in indium (99.9995% purity) mounts using a hydraulic press (*ca.* 5 tonn) then covered with a gold coat *ca.* 30 nm thick. The oxygen isotope data were acquired at the University of Edinburgh using a Cameca ims 1270. A ^133^Cs^+^ focussed primary ion beam of ∼4 nA was rastered over an area of 25 μm^2^. Secondary ions were extracted at 10 kV and ^16^O, ^17^O and ^18^O were monitored at a mass resolution of ∼6000 using either a Faraday cup or an electron multiplier depending on the count rates of the isotope. Background, relative detector yield and dead-time corrections were applied to the count-rates recorded. Each analysis involved a pre-sputtering time of 60 s, followed by automatic centring of the secondary ion beam into the field aperture (3000 μm) and entrance slits (30 μm). Isotopic data was acquired in two blocks of ten cycles, amounting to a total count time of 80 seconds per isotope. Results were obtained with standard error of the mean in the range 0.3–6% depending on the uniformity of the sample surface. Instrument calibration and alignment was checked by measuring the isotopic ratios of a natural abundance mineral standard.

## Results and discussion

**Fig. 2:**
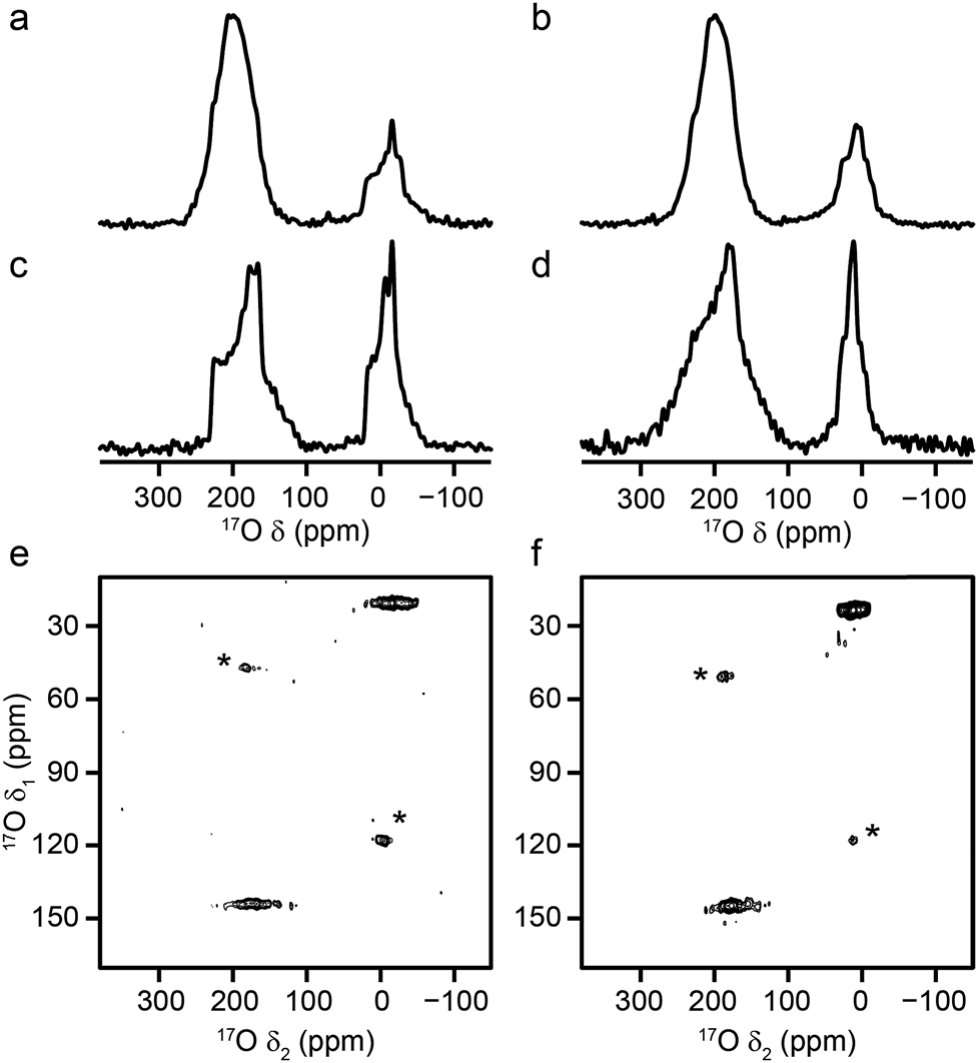
^17^O (14.1 T, 20 kHz) spin echo MAS NMR spectra of (a, b) as-made and (c, d) calcined samples of (a, c) Al MIL-53 and (b, d) Ga MIL-53. ^17^O (14.1 T, 20 kHz) MQMAS NMR spectra of calcined (e) Al MIL-53 and (f) Ga MIL-53. Asterisks denote spinning sidebands.

Al and Ga MIL-53 materials were synthesised using DGC, and were shown by mass spectrometry to have an ^17^O enrichment level ranging from 11–21%, as described in the ESI.† Fig. 2 shows ^17^O MAS NMR spectra of the as-made and calcined forms of Al and Ga MIL-53 (the corresponding ^1^H MAS, ^13^C CP MAS and ^27^Al MAS NMR spectra are shown in the ESI†). Two broadened ^17^O resonances are observed in the as-made forms. The lineshapes sharpen upon calcination, and display features characteristic of second-order quadrupolar broadening. The signal at higher shift can be assigned to carboxylate oxygens^36^ in the terephthalate linker (as this signal is not observed in ^1^H–^17^O CP experiments, not shown). The signal at lower shift results from the hydroxyl groups that bridge between the metal centres in this material.^36^ Although there appears to be a relative loss (of ∼40%) of ^17^O signal for the carboxylate group upon calcination, this is a result of the loss of the excess free linker (also enriched by the synthetic process) found within the pores. There is some decomposition observed for the Ga MIL-53 sample, leading to a broader component in the spectral lineshapes.^57^ There is a clear difference in the position of the hydroxyl resonances for the Al- and Ga-containing materials, with these centred at –6.6 and 14.2 ppm, respectively; evidence of the sensitivity of ^17^O NMR to the type of metal cations within the framework. ^17^O MQMAS spectra of the calcined materials are also shown in Fig. 2. It is possible to obtain values for the NMR parameters (*δ*_iso_ and *P*_Q_) for the two types of chemical species from the positions of the centres-of-gravity of the spectral lineshapes, which are given in Table 1. For Al MIL-53, it is also possible to fit the lineshapes in the ^17^O MAS spectrum to obtain estimates of *C*_Q_ and *η*_Q_ (also given in Table 1). These parameters reveal that the largest differences upon substitution of the metal centre are not in the changes to *δ*_iso_, but are in the magnitude of the quadrupolar coupling (with *P*_Q_ for the hydroxyl O of 6.0 MHz for Al MIL-53 and 4.6 MHz for Ga MIL-53).

**Table 1:** NMR parameters obtained from the ^17^O MQMAS NMR spectra shown in Fig. 2 and 3

O species	*δ*_iso_ (ppm)	*P*_Q_/MHz	*C*_Q_/MHz	*η*_Q_
**Calcined Al MIL-53**				
Carboxylate	230(3)	7.8(3)	7.1(2)	0.8(2)
Hydroxyl	20(3)	6.0(3)	5.5(2)	0.7(2)

**Calcined Ga MIL-53**				
Carboxylate	230(3)	7.7(3)		
Hydroxyl	31(3)	4.6(4)		

**Calcined, hydrated Al MIL-53** ^*a*^				
Carboxylate 1	255(3)	7.8(3)	7.1(3)	0.7(1)
Carboxylate 2	225(5)	8.5(3)	7.9(3)	0.7(1)
Hydroxyl	15(5)	7.2(5)	5.8(5)	0.9(1)

**Calcined, hydrated Ga MIL-53** ^*a*^				
Carboxylate 1	259(2)	7.5(2)		
Carboxylate 2	230(5)	8.1(5)		
Hydroxyl	24(6)	5.9(8)		

**Calcined, hydrated Al, Ga (50** **:** **50) MIL-53**^*a*^				
Carboxylate 1	253(3)	8.0(3)		
Carboxylate 2	225(3)	8.5(3)		
Hydroxyl	12(3)	7.2(3)		

**Calcined, hydrated Al, Ga (80** **:** **20) MIL-53**^*a*^				
Carboxylate 1	256(3)	7.8(3)		
Carboxylate 2	224(3)	8.6(3)		
Hydroxyl	10(3)	7.5(3)		

^*a*^Note that for the hydrated materials the parameters are average values only, as a result of the disorder.

**Fig. 3:**
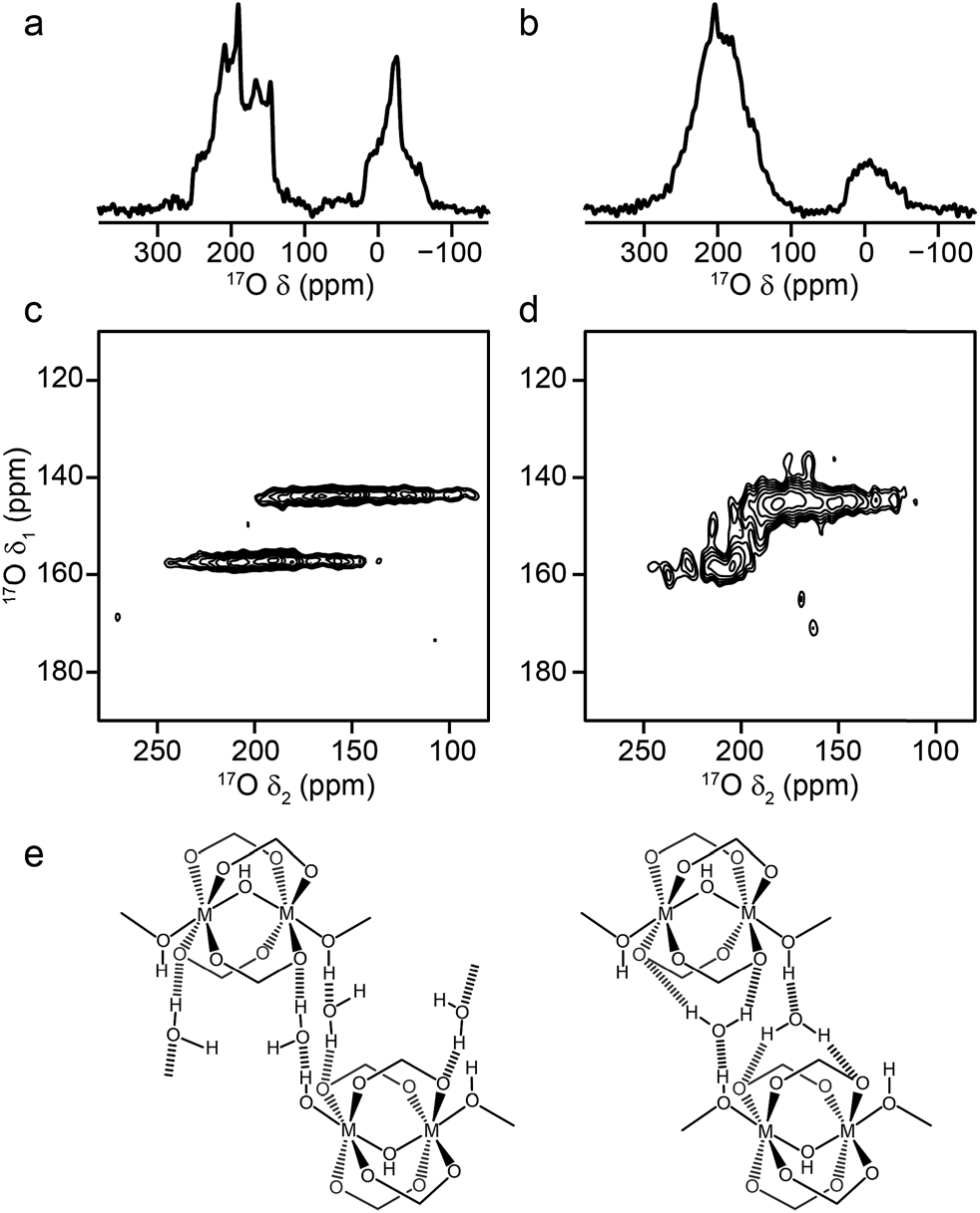
^17^O (14.1 T, 20 kHz) (a, b) spin echo MAS and (c, d) MQMAS NMR spectra of calcined, hydrated (a, c) Al MIL-53 and (b, d) Ga MIL-53. In (c, d), only the carboxylate region of the spectrum is shown. (e) Possible hydrogen-bonding arrangements in hydrated MIL-53 (M = Al, Ga) based on DFT-optimised literature^59^ structures. See ESI for more detail.†

A significant change is observed in the ^17^O MAS NMR spectrum of Al MIL-53 upon hydration,^36^ as shown in Fig. 3a. The ^17^O MQMAS spectrum (Fig. 3c) resolves two distinct sites in the carboxylate region of the spectrum, and demonstrates the sensitivity of ^17^O NMR spectroscopy to the changes in local geometry that result from the change in the pore size and shape, highlighted in Fig. 1. Average NMR parameters, extracted from the centres of gravity of the spectral lineshapes, are given in Table 1. The observation of two distinct carboxylate oxygens in the MQMAS spectrum supports prior computational^8^ and structural studies^57^–^59^ of different hydrogen-bonding interactions of adsorbed water in the pores of the framework. This work suggested the presence of two types of channels in the hydrated structures of Al and Ga MIL-53, where the two inequivalent water molecules are characterised by different hydrogen bonding schemes. Density functional theory (DFT) calculations were carried out in order to investigate whether this phenomenon could also lead to two sets of inequivalent carboxylate ^17^O species. On geometry optimisation of the literature structures of hydrated Al and Ga MIL-53 (ref. 59) (**A′**_**Al**_ and **B′**_**Ga**_, respectively), the encapsulated water molecules moved significantly to form hydrogen-bonding interactions with the carboxylate oxygen centres. Exchanging Al and Ga between the two optimised structures and reoptimising (to give **B′**_**Al**_ and **A′**_**Ga**_) showed that both structural types are energetically accessible (see ESI for details†). DFT-predicted NMR parameters based on the optimised geometries (see Fig. 3e and ESI†) allowed assignment of the ^17^O NMR spectra where ^17^O species at *δ*_iso_ = 225 ppm are hydrogen bonded to water whereas those with *δ*_iso_ = 255 ppm are not involved in hydrogen bonding. The oxygen centres involved in hydrogen bonding are dictated by the distortion of the framework on narrowing, which presumably allows the carboxylate ^17^O species to remain inequivalent during any motion of the water. Notably, the optimised structures **B′**_**Al**_ and **B′**_**Ga**_ exhibit equivalent water molecules and channels while retaining two inequivalent sets of carboxylate oxygen species.

**Fig. 4:**
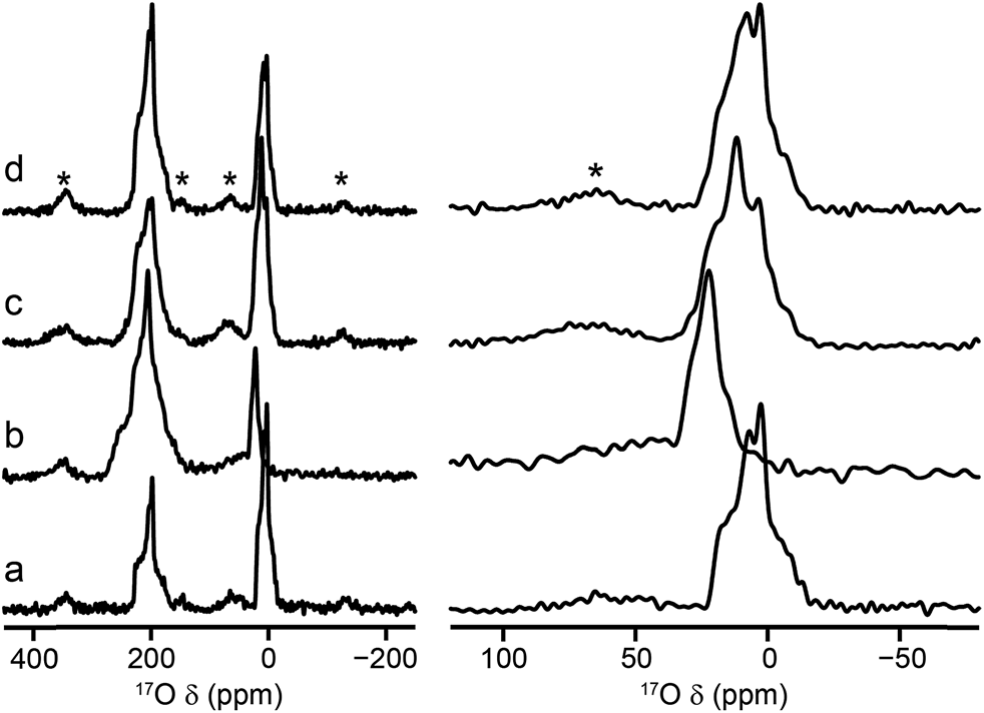
^17^O (20.0 T, 16 kHz) spin echo MAS NMR spectra of calcined (a) Al, (b) Ga, (c) Al, Ga (50 : 50, stoichiometric ratio) and (d) Al, Ga (80 : 20, stoichiometric ratio) MIL-53, with an expansion of the hydroxyl region shown on the right. Asterisks denote spinning sidebands.

**Fig. 5:**
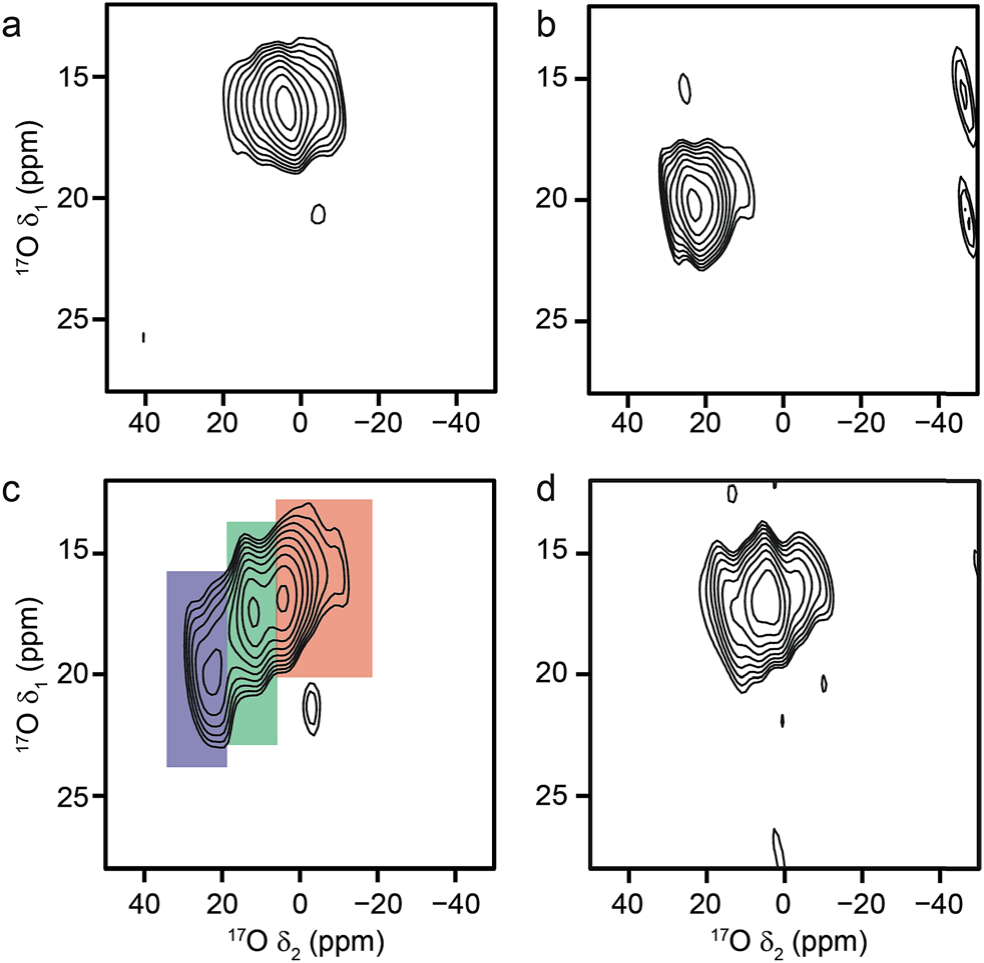
^17^O (20.0 T, 20 kHz) MQMAS spectra of calcined (a) Al, (b) Ga, (c) Al, Ga (50 : 50, stoichiometric ratio) and (d) Al, Ga (80 : 20, stoichiometric ratio) MIL-53. Signals corresponding to hydroxyls bridging Al/Al, Ga/Ga and Al/Ga centres are highlighted in red, blue and green, respectively.

Mixed-metal MIL-53 materials were produced by using different stoichiometric ratios (Al : Ga of 50 : 50 and 80 : 20) of the two metal nitrate sources in the synthesis (see ESI†). ^17^O MAS NMR spectra of the calcined forms of these materials are compared with those for the Al and Ga end members in Fig. 4. Although the carboxylate lineshapes appear similar, there are differences in the hydroxyl region of the spectra, as shown in the expansion on the right in Fig. 4. This is seen more clearly in the ^17^O MQMAS spectra in Fig. 5, where the increased resolution shows that the spectrum for the 50 : 50 material is not simply the sum of the lineshapes seen for the two end members, but contains an additional, distinct resonance. This clearly indicates the presence not only of hydroxyls that bridge between two Al (as in Al MIL-53) and two Ga (as in Ga MIL-53), but hydroxyls that bridge between Al and Ga, *i.e.*, an atomic-level mixing of the metal cations. The average quadrupolar parameters extracted from the spectra in Fig. 5 are shown in Table 2. For the 80 : 20 mixed-metal material the MQMAS spectrum is dominated by the signal seen in the Al end member, but it is clear from the MAS spectra that the two other resonances are also present.

**Table 2:** NMR parameters for the hydroxyl ^17^O species obtained from the MQMAS NMR spectra shown in Fig. 5

	*δ*_iso_ (ppm)	*P*_Q_/MHz
**Calcined Al MIL-53**		
Hydroxyl	20(3)	6.0(3)

**Calcined Ga MIL-53**		
Hydroxyl	32(3)	4.4(3)

**Calcined Al, Ga (50** **:** **50) MIL-53**		
Hydroxyl 1	21(3)	6.1(3)
Hydroxyl 2	24(3)	5.2(3)
Hydroxyl 3	31(3)	4.3(3)

**Calcined Al, Ga (80** **:** **20) MIL-53**		
Hydroxyl	21(3)	6.1(3)

**Fig. 6:**
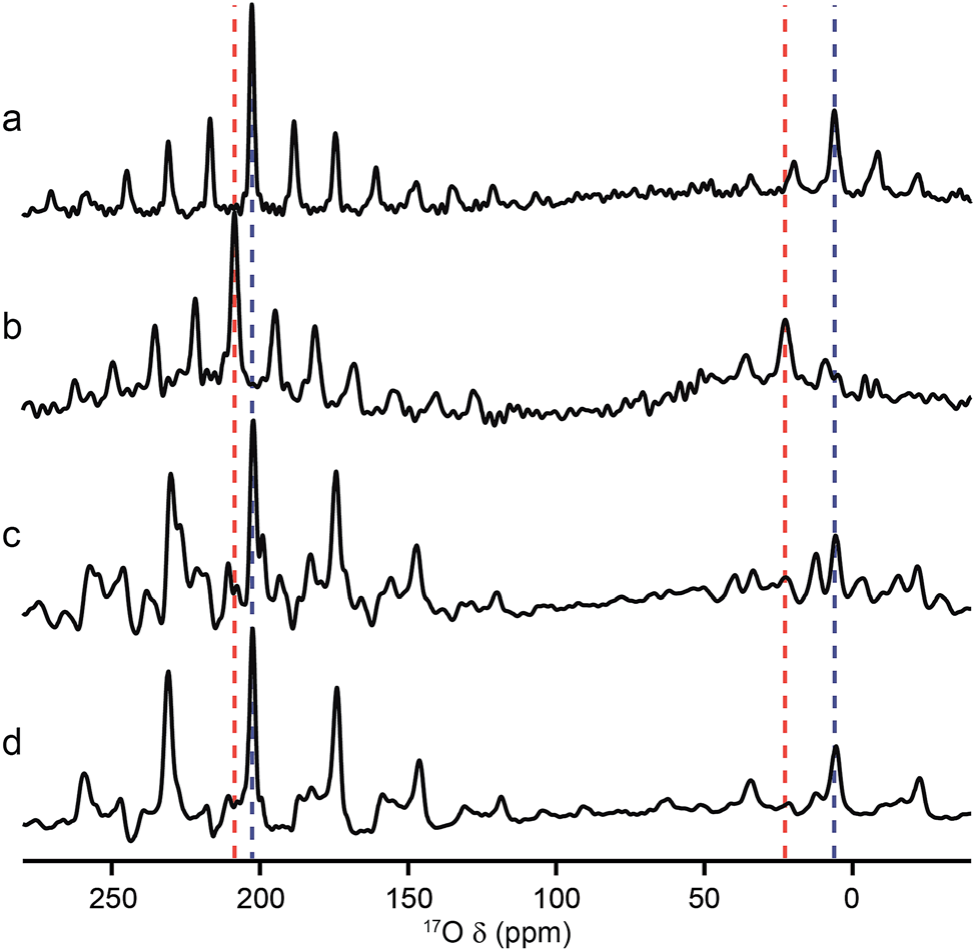
^17^O (20.0 T) DOR spectra of calcined (a) Al MIL-53, (b) Ga MIL-53, (c) Al, Ga (50 : 50) MIL-53 and (d) Al, Ga (80 : 20) MIL-53. MAS rates were (a) 1.6/7.7 kHz, (b) 1.55/7.9 kHz, (c) 1.63/7.9 kHz and (d) 1.65/8.0 kHz. Dashed lines indicate the position of the isotropic resonances for Al (blue) and Ga (red) MIL-53.

Although MQMAS spectra can provide high resolution, they are inherently non quantitative, owing to the dependence of the efficiency of multiple-quantum filtration on the quadrupolar coupling. The single-quantum DOR (double rotation) experiment, in which the sample is rotated about two angles simultaneously to remove the quadrupolar broadening, can provide an alternative approach. However, the experiment is technically challenging and only slow rotation rates (between 1.2 and 1.8 kHz for the outer rotor and 6–8 kHz for the inner rotor) can be achieved. Experiments are, therefore, typically performed at two different spinning speeds to identify the isotropic centre bands from the many spinning sidebands. Fig. 6 shows ^17^O DOR spectra of the end member and mixed-metal MOFs, with the position of the isotropic resonances for the two end members highlighted. In each case, corresponding spectra acquired with variable spinning rates are shown in the ESI.† For the two end members (Fig. 6a and b) a single isotropic resonance is seen in both the carboxylate and hydroxyl regions of the spectrum (at ∼202 and ∼6 ppm for Al MIL-53 and at ∼208 and ∼22 ppm Ga MIL-53). For Al, Ga (50 : 50) MIL-53, as shown in Fig. 6c, peaks are observed at 6 and 22 ppm in the hydroxyl region, in good agreement with the positions of the peaks observed for the two end members (note that the peak at 22 ppm is overlapped with a spinning sideband and appears distorted). However, a third resonance is also clearly observed at 12 ppm, resulting from hydroxyls bridging Al and Ga centres. Similarly, in the carboxylate region signals are seen at 208 and 202 ppm, reflecting the position of the resonances seen in the two end members (202 ppm for Al and 208 ppm for Ga), and at 199 ppm for the mixed-metal material. A similar result is obtained for Al, Ga (80 : 20) MIL-53, although the peaks corresponding to carboxylates and hydroxyls bridging two Al species are significantly more intense.

^17^O NMR spectroscopy clearly shows that Al and Ga are intimately mixed in the Al, Ga MIL-53 materials, *i.e.*, that the materials do not comprise a two-phase mixture of Al MIL-53 and Ga MIL-53. This conclusion is also supported by ^27^Al NMR experiments (shown in the ESI†), where MQMAS spectra reveal a distribution of ^27^Al parameters in calcined Al, Ga MIL-53 materials. This confirms a range of local environments is present (unlike in the Al end member), arising from differing numbers and levels of Ga substitution in nearby cation sites.

**Fig. 7:**
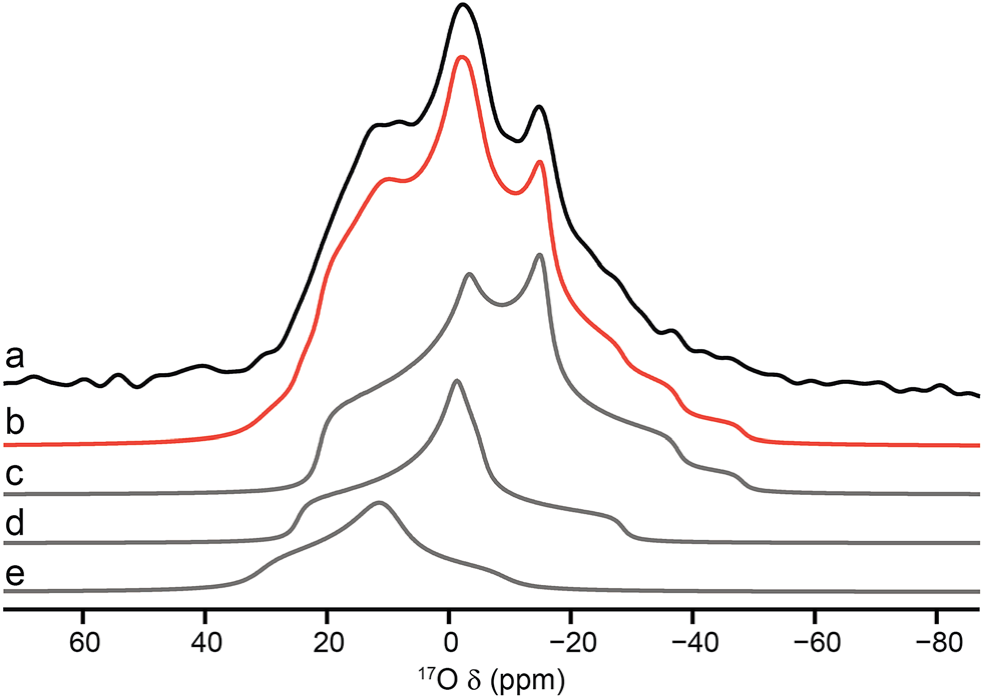
(a) ^17^O (14.1 T, 20 kHz) MAS NMR spectrum of calcined Al, Ga (50 : 50) MIL-53 (hydroxyl region only). Overall fit (b) and decomposition into components resulting from hydroxyls that bridge between (c) Al/Al, (d) Al/Ga and (e) Ga/Ga.

Using the quadrupolar parameters extracted from ^17^O MQMAS spectra, it is possible to fit ^17^O MAS spectra (acquired using a short flip angle to ensure quantitative relative intensities were obtained) of the mixed materials to determine the proportion of each species present. Attention was focussed only on the hydroxyl region of the spectrum because of the higher resolution exhibited. An example is shown in Fig. 7, for calcined Al, Ga (50 : 50) MIL-53. The proportions of each component for the two mixed-metal MOFs are given in Table 3. Note that although isotropic resonances are obtained in DOR spectra, the presence of many sidebands, and their overlap with some of the centre bands, makes it impossible to extract accurately the proportion of each signal directly from these spectra. However, the spectra shown in Fig. 6 are consistent with the results shown in Table 3. The relative proportions of the three signals suggest actual compositions of Al, Ga MIL-53 of 70 : 30 and 92 : 8 for the samples with nominal (*i.e.*, starting) compositions of 50 : 50 and 80 : 20, respectively. This suggests that not all the Ga used is incorporated into the final product, although it should be noted that the mass of the hydrated nitrate reagent is not known exactly. Furthermore, the Ga that is incorporated is not randomly distributed in the materials. If this were the case, 42% of the hydroxyls would bridge two different metal centres for a material with 70 : 30 composition (and 15% for one with 92 : 8). Table 3 shows that only 26% (and 9%) of hydroxyls link Al and Ga, indicating preferential clustering of like atoms.

**Table 3:** Relative proportions of the bridging hydroxyl oxygen species and the Al : Ga ratio of calcined mixed-metal MIL-53, determined from short flip angle ^17^O MAS NMR spectra

	Relative intensity (%)	
	Al, Ga (80 : 20)	Al, Ga (50 : 50)
Al/Al	88(1)	58(1)
Ga/Ga	3(1)	16(1)
Al/Ga	9(1)	26(1)
Actual Al : Ga	92 : 8	70 : 30

Rietveld refinements of powder XRD patterns of the as-made materials confirm that two types of metal cations are present. Although not the ideal method to determine composition, the refinements are consistent with an Al/Ga ratio of 70 : 30 and 78 : 22 for the two mixed-metal MOFs (although the uncertainty on these values is high), and the unit cell size (obtained through Pawley fitting) was increased relative to that found for the Al end member,^8^ consistent with Ga substitution (see ESI†). Further support is also obtained using EDX, where average compositions of 72 ± 9 : 28 ± 9 and 84 ± 11 : 16 ± 11 were obtained for the two materials (see ESI†), again in good agreement with the values determined using NMR spectroscopy.

**Fig. 8:**
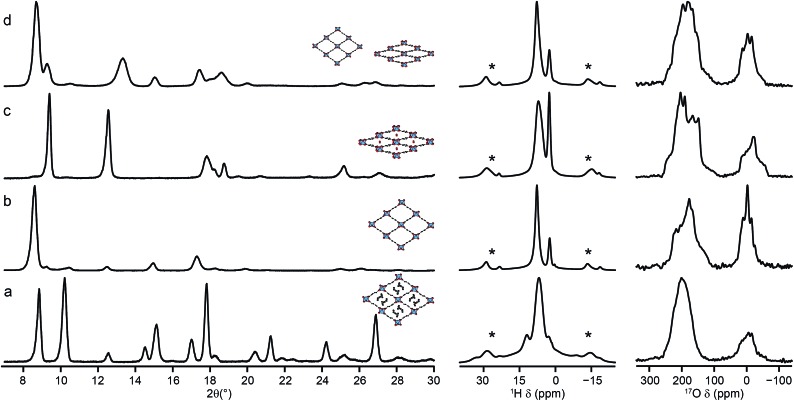
Powder XRD patterns (and schematic structures), ^1^H (14.1 T, 12.5 kHz) and ^17^O (14.1 T, 20 kHz) MAS NMR spectra of Al, Ga (50 : 50) MIL-53 in its (a) as-made, (b) calcined open-pore, (c) hydrated closed-pore and (d) dehydrated mixed-pore forms. Asterisks denote spinning sidebands.

The reversible “breathing” behaviour of MIL-53, and the resulting large variations in pore size, has led to considerable interest in the material. As shown in Fig. 1, for Al MIL-53 the as-made form converts to a large-pore form upon calcination, which can then be reversibly converted to the narrow-pore hydrated form. This can be clearly seen by powder XRD (shown in the ESI†). However, Ga MIL-53 remains in the closed-pre form upon dehydration.^14^,^56^ The XRD patterns also show the partial decomposition of Ga MIL-53 upon calcination, as discussed above, indicating its lower thermal stability. Powder XRD patterns of as-made, calcined, hydrated and dehydrated Al, Ga (50 : 50) MIL-53, shown in Fig. 8, reveal a very different breathing behaviour for this material. In all forms, the unit cell size is larger than that of Al MIL-53,^8^ confirming substitution of Ga (see ESI†). The mixed-metal MIL-53 showed no evidence of decomposition upon calcination, unlike Ga MIL-53. However, when the rehydrated calcined material was subsequently dehydrated, a mix of narrow-pore and open-pore forms was obtained (see Fig. 8). A Pawley fit (see ESI†) suggests that the closed-pore form is slightly distorted compared to the closed pores seen in the hydrated form. The ^1^H MAS NMR spectra (also shown in Fig. 8) show the loss of signals (*e.g.*, ∼11 ppm) from excess BDC upon calcination and the growth of signals from water upon hydration (overlapped with the hydroxyl at 2.6 ppm and with signal from the linker molecules).^8^ The ^1^H spectrum for the dehydrated form is similar to that of the calcined form, but it is clear from the ^17^O MAS spectrum (Fig. 8d) that a mixed-, rather than purely open-pore form is present. It is clear in ^17^O MQMAS NMR spectra of the hydrated and dehydrated forms of Al, Ga MIL-53 (see ESI†), that the closed pores found in the dehydrated form are different from those in the hydrated form (as noted above by XRD), with a distinct change in the position of the carboxylate resonance at highest shift. As discussed above, EDX confirmed all crystallites contain both Al and Ga, although some deviate significantly from the average composition, accounting for the relatively large standard deviation reported. This suggests that different crystallites may adopt different forms dependent on their Al and Ga content.

**Fig. 9:**
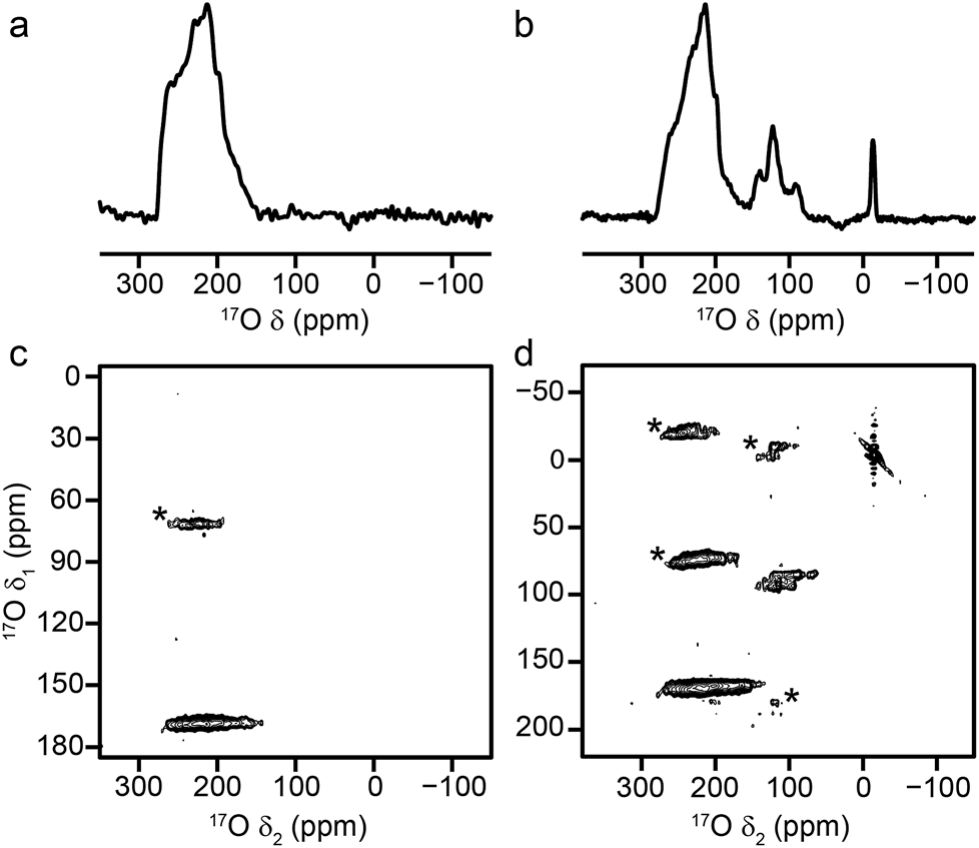
^17^O (14.1 T, 20 kHz) (a, b) spin echo MAS and (c, d) MQMAS NMR spectra of (a, c) calcined Sc_2_(BDC)_3_ and (b, d) as-made steamed Sc MIL-53. Asterisks denote spinning sidebands.

Attempts to prepare Sc MIL-53 also using DGC under the conditions used for Al- and Ga-containing samples produced a different terephthalate MOF, Sc_2_BDC_3_ (confirmed using powder XRD and solid-state NMR as described in the ESI†). This small-pore MOF has two crystallographically-distinct linkers (in a 2 : 1 ratio),^12^,^60^–^62^ but has no hydroxyl groups. The ^17^O MAS NMR spectrum of calcined Sc_2_BDC_3_, shown in Fig. 9a, confirms the lack of a hydroxyl O signal. The MQMAS spectrum is not able to resolve the two ^17^O resonances expected in the carboxylate region owing to the presence of two distinct linker molecules, suggesting that these are very similar. NMR parameters determined from the position of the resonance in the MQMAS spectrum are given in Table 4, and show that *C*_Q_ is similar to that seen for carboxylate species in the Al and Ga MIL-53 materials, but that *δ*_iso_ is significantly different.

**Table 4:** NMR parameters obtained from the ^17^O MQMAS NMR spectra shown in Fig. 9

O species	*δ*_iso_ (ppm)	*P*_Q_/MHz
**Calcined Sc**_**2**_ **(BDC)**_**3**_		
Carboxylate	273(3)	8.1(3)

**Steamed (hydrated) Sc MIL-53**		
Carboxylate	272(3)	8.1(3)
Hydroxyl	143(4)	6.0(4)

The attempted DGC synthesis of a mixed-metal (Sc, Al) form of MIL-53 resulted in a two-phase sample containing Sc_2_BDC_3_ and Al MIL-53. Although the presence of very small amounts of cation substitution in either phase cannot be discounted completely, the powder XRD pattern and ^1^H/^13^C NMR spectra appear to contain a combination of signals that match those obtained for the two pure phases (see ESI†). The ^17^O MAS and MQMAS NMR spectra (also shown in the ESI†) confirm the presence of resonances from the two separate phases. Although Sc_2_(BDC)_3_ has a lower metal : linker ratio than MIL-53, decreasing the amount of terephthalic acid used (or varying the reaction temperature) did not lead to the formation of MIL-53, with all Sc incorporated into a BDC phase in each case. Therefore, Sc MIL-53 was prepared using conventional solvothermal synthesis,^12^ and post-synthetic ^17^O enrichment attempted using a steaming procedure. The ^17^O MAS and MQMAS spectra of Sc MIL-53 shown in Fig. 9b and d confirm successful enrichment (estimated to be ∼25% by comparison to previous materials), and formation of the MIL-53 framework structure. The pores contain water exchanged during steaming process, giving what has been termed in the literature as the “intermediate” phase,^12^ and leading to the sharp signal at ∼13 ppm in the spectrum. Resonances from both carboxylate and hydroxyl species are observed, although with different chemical shifts to those for the Al and Ga end members (as given in Table 4). Clearly, post-synthetic steaming offers an alternative, and potentially very useful, approach to ^17^O enrichment for MOFs that cannot be prepared directly by DGC. We will explore this method for the synthesis of further mixed-metal materials in more detail in future work.

## Conclusions

Using the atom-efficient DGC synthesis approach we have been able to enrich Al and Ga MIL-53 MOFs with ^17^O. As the synthesis containing Sc formed the small-pore MOF Sc_2_(BDC)_3_, Sc MIL-53 was enriched using a novel post-synthetic steaming of a sample prepared solvothermally. Mass spectrometry shows that the samples prepared by DGC had enrichment levels of between 11 and 21% (at a cost of *ca.* €750 per g of as-made MOF). A recent study^63^ involving an economic mechanochemical ^17^O enrichment of strategic precursors, such as carboxylic acids, achieved enrichment levels of 3.5% in 2 h with a lower cost of €220 per g of linker, for BDC, for example. However, during hydrothermal or dry gel conversion conditions back exchange of the enriched linker is likely to occur, potentially lowering the level of isotopic enrichment of the final product and making the enrichment of the starting linker a less favourable approach for more complex materials.

^17^O MAS spectra of MIL-53 resolve resonances from carboxylate and hydroxyl species and are very sensitive both to changes in pore structure (*e.g.*, upon adsorption of water) and to cation substitution. MQMAS spectra provide resolution of the two types of ^17^O carboxylates upon hydration. DFT calculations based on the literature structures of hydrated Al and Ga MIL-53 (ref. 59) show that the two inequivalent carboxylate ^17^O species result from distortion of the pores upon narrowing, giving two short and two long contacts to the bridging hydroxyl. Water is found to preferentially bridge the shorter OH···O contact through hydrogen-bonding interactions. The two different models resulting from optimisation of the literature structures have very similar DFT energies for both Al and Ga forms of MIL-53.

For Al, Ga mixed-metal MIL-53 materials, high-resolution ^17^O NMR spectroscopy (using MQMAS and DOR experiments) proves the presence of hydroxyl groups linked to one Al and one Ga centre, confirming the formation of a true mixed-metal material rather than a simple two-phase mixture. However, the ratio of the different spectral resonances in this region in a quantitative spectrum suggests that the Al : Ga ratio is different from that in the initial synthesis mixture, *e.g.*, a nominal ratio of 50 : 50 in the reaction mixture gave a final product with 70 : 30. This was also supported by powder XRD and EDX. Furthermore, the MAS NMR spectra confirmed that the Ga is not randomly distributed in the material, but that there is some clustering (falling short of real ordering) of like atoms within the materials.

The incorporation of Al into Ga MIL-53 reduced thermal decomposition of the material upon calcination. Unlike the two single-metal materials (where dehydration leads to a large-pore form in Al MIL-53 and narrow-pore form in Ga MIL-53), dehydration of the mixed-metal MOF produced a mix of closed-pore and open-pore forms, most likely reflecting the adoption of a different structural form for crystallites with differing Al : Ga ratios. This perhaps offers the intriguing future possibility of dual-adsorption behaviour for some guest molecules in mixed-metal MIL-53 if the proportion and distribution of cations can be controlled.

The DGC enrichment process did not yield Sc MIL-53, but did produce ^17^O-enriched Sc_2_(BDC)_3_. Enriched Sc MIL-53 was successfully produced using post-synthetic steaming, where the solveothermally pre-synthesised MOF is heated with H_2_^17^O vapour. This provides a relatively cost- and atom-efficient route to enriched MOFs, removing the need to significantly adapt previously optimised synthetic procedures. In future work, we hope to evaluate the wider applicability of this approach, and use this to investigate cation disorder and its effect on the physical and chemical properties of MOFs.

## Conflicts of interest

There are no conflicts of interest to declare.

## Supplementary Material

Supplementary information
