# Supplementary material for: Cost-effective 17O enrichment and NMR spectroscopy of mixed-metal terephthalate metal–organic frameworks
Source: Chem Sci. 2017 Nov 23;9(4):850–9. doi: 10.1039/c7sc04649a (PMC5873045; doi:10.1039/c7sc04649a)
Supplement: Supplementary file 1 [file SC-009-C7SC04649A-s001.pdf]

Electronic Supplementary Material (ESI) for Chemical Science.
This journal is © The Royal Society of Chemistry 2017

Cost-Effective ^{17}O Enrichment and NMR Spectroscopy of Mixed-Metal Terephthalate Metal-Organic Frameworks

Giulia P. M. Bignami, Zachary H. Davis, Daniel M. Dawson, Samuel A. Morris, Samantha E. Russell, David McKay, Richard E. Parke, Dinu Iuga, Russell E. Morris and Sharon E. Ashbrook

Supporting Information

S1. Synthetic procedures

S2. Mass spectrometry

S3. NMR spectra of Al MIL-53 and Ga MIL-53

S4. DFT calculations on rehydrated Al MIL-53 and Ga MIL-53

S5. NMR spectra of Al,Ga MIL-53

S6. Powder XRD, EDX and SEM measurements

S7. Characterisation of $Sc_2(BDC)_3$

S8. Characterisation of mixed-phase Al MIL-53 and $Sc_2(BDC)_3$

S9. Additional ^{17}O experimental NMR parameters

S10. References

S1. Synthetic procedures

Dry gel conversion (DGC) syntheses were carried out using the reagents as described in Table S1.1, following the method in Ref. S1, as outlined in the main text. For DGC syntheses using $Sc(NO_3)_3$, the product formed was shown to be (partly or wholly) Sc_2BDC_3 rather than the intended MIL-53. In these cases, Sc-containing MIL-53 materials were synthesised *via* a solvothermal route (as described in Ref. S2 and discussed in the main text), and subsequently enriched using a post-synthesis steaming approach.

Table S1.1 Molar quantities of reagents used for DGC syntheses. In cases where the water content of the nitrate salt was not known exactly the weight was determined using the anhydrous molar mass.

$Al(NO_3)_3.9H_2O$ / mmol	$Ga(NO_3)_3.xH_2O$ / mmol	$Sc(NO_3)_3.xH_2O$ / mmol	H_2BDC / mmol	Product[a]
1.38			1.81	Al MIL-53
	1.38		1.81	Ga MIL-53
0.69	0.69		1.81	(Al,Ga) MIL-53 (50:50)
1.10	0.28		1.81	(Al,Ga) MIL-53 (80:20)
0.69		0.69	1.81	Al MIL-53 + $Sc_2(BDC)_3$
		1.38	1.81	$Sc_2(BDC)_3$

[a] In some cases the product was subsequently demonstrated to have a chemical composition that differed to that intended

S2. Mass spectrometry

Samples were studied using mass spectrometry, as described in the main text. Results are reported below as counts per isotope, with the instrumental standard error of the mean per measurement. The final percentage of ^{17}O is obtained by comparison to the natural abundance $^{16}O/^{17}O$ ratio, and is reported as the interval determined by the standard deviation calculated on the average of all measurements.

Table S2.1 O isotopes in the standard $FeTiO_3$ (ilmenite) sample, used as a reference. The amount of ^{17}O detected is 0.035-0.036%.

^{16}O (counts)	% standard error	^{17}O (counts)	% standard error	^{18}O (counts)	% standard error
4.20×10^8	3.1	1.49×10^5	3.0	7.50×10^5	3.1
5.14×10^8	0.3	1.83×10^5	0.2	9.38×10^5	0.2
5.12×10^8	0.1	1.82×10^5	0.1	9.32×10^5	0.1

Table S2.2 O isotopes in natural-abundance Al MIL-53, prepared by DGC. The amount of ^{17}O detected is 0.034-0.035%.

^{16}O (counts)	% standard error	^{17}O (counts)	% standard error	^{18}O (counts)	% standard error
6.18×10^7	4.9	2.17×10^4	4.9	1.07×10^5	5.0
2.30×10^7	13.0	7.93×10^3	13.0	3.80×10^4	13.0
1.44×10^7	13.0	4.93×10^3	13.0	2.39×10^4	13.0

Table S2.3 O isotopes in as-made ^{17}O-enriched Al MIL-53, prepared by DGC. The amount of ^{17}O detected is 15-20%.

^{16}O (counts)	% standard error	^{17}O (counts)	% standard error	^{18}O (counts)	% standard error
8.34×10^7	1.8	1.59×10^7	1.9	2.59×10^5	1.7
1.21×10^7	5.4	2.15×10^6	5.4	3.51×10^4	6.7
3.49×10^7	1.3	8.57×10^6	1.7	1.26×10^5	1.5
1.02×10^8	0.3	1.86×10^7	0.4	3.12×10^5	0.3
1.27×10^8	0.7	2.31×10^7	1.3	3.98×10^5	1.0

Table S2.4 O isotopes in a calcined hydrated ^{17}O-enriched Al,Ga MIL-53, prepared by DGC. The amount of ^{17}O detected is 16-21%.

^{16}O (counts)	% standard error	^{17}O (counts)	% standard error	^{18}O (counts)	% standard error
3.83×10^7	2.0	8.55×10^6	3.4	2.94×10^5	3.0
2.99×10^7	0.6	6.20×10^6	1.1	2.21×10^5	0.9
3.67×10^7	1.3	9.24×10^6	1.2	3.12×10^5	1.2
3.38×10^7	0.8	8.83×10^6	1.1	3.01×10^5	1.0
3.78×10^7	2.5	7.38×10^6	4.5	2.62×10^5	4.0

Table S2.5 O isotopes in a calcined ^{17}O-enriched Al MIL-53, prepared by DGC. The amount of ^{17}O detected is 11-16%.

^{16}O (counts)	% standard error	^{17}O (counts)	% standard error	^{18}O (counts)	% standard error
5.87×10^{5}	4.1	7.36×10^{4}	4.8	3.17×10^{3}	4.6
3.98×10^{7}	1.5	6.49×10^{6}	2.0	2.44×10^{5}	1.7
1.64×10^{7}	7.3	3.24×10^{6}	7.4	1.13×10^{5}	7.6
3.15×10^{7}	4.4	5.02×10^{6}	4.6	1.87×10^{5}	4.5
3.63×10^{7}	2.7	6.63×10^{6}	1.8	2.39×10^{5}	2.0
3.44×10^{7}	6.2	5.48×10^{6}	5.9	2.02×10^{5}	6.1

S3. NMR spectra of Al MIL-53 and Ga MIL-53

^{1}H MAS and ^{13}C CP MAS NMR spectra of as-made and calcined Al MIL-53 and Ga-MIL-53 are shown in Figures S3.1 and S3.2. Also shown in Figures S3.3 and S3.4 are ^{27}Al MAS and MQMAS NMR spectra of Al MIL-53.

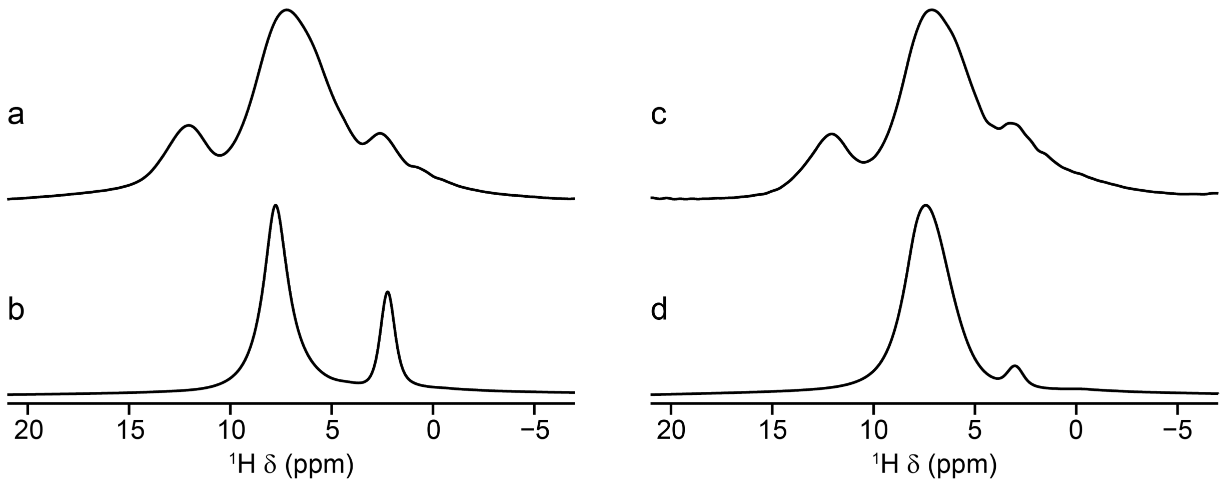

Figure S3.1 ^{1}H (14. T, 12.5 kHz) MAS NMR spectra of (a, c) as-made and (b, d) calcined samples of (a, b) Al MIL-53 and (c, d) Ga MIL-53. The spectrum in (c) was acquired using a spin echo.

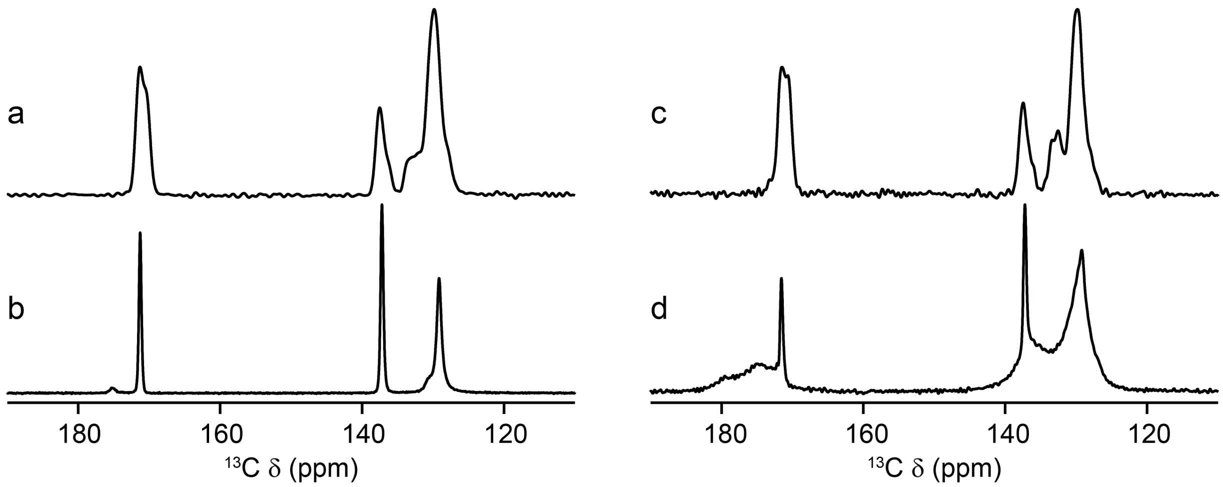

Figure S3.2 ^{13}C (14.1 T, 12.5 kHz) CP MAS NMR spectra of (a, b) as-made and calcined Al MIL-53 and (c, d) as-made and calcined Ga MIL-53.

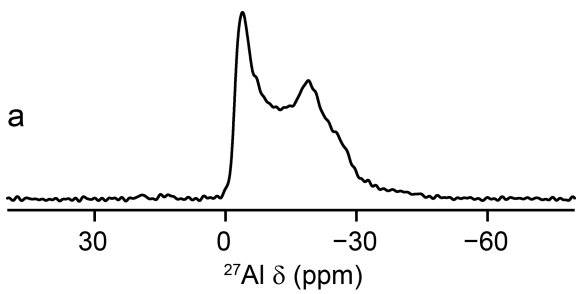

Figure S3.3 ^{27}Al (14.1 T) MAS NMR spectra of (a) as-made (MAS rate of 16 kHz) and (b) calcined Al MIL-53 (MAS rate of 14 kHz), acquired using a short flip angle.

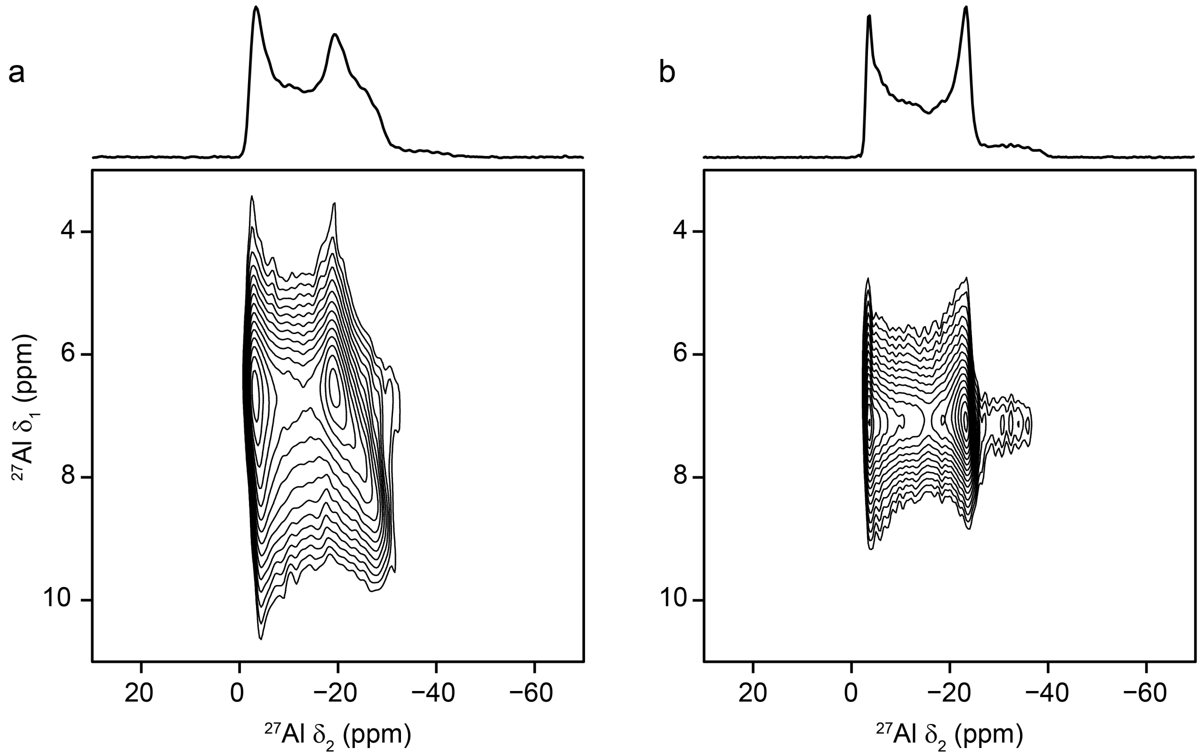

Figure S3.4 ^{27}Al (14.1 T) MQMAS NMR spectra of (a) as-made (MAS rate of 16 kHz) and (b) calcined Al MIL-53 (MAS rate of 14 kHz).

For as-made Al MIL-53, the ^{27}Al MQMAS spectrum shows a distribution of NMR parameters, indicating disorder in the local structure. This arises from the presence of excess disordered linker molecules in the pores. Fitting of cross sections parallel to δ_2 shows that there is a variation in C_Q from ~7.8 to ~9.1 MHz, but a much smaller variation in δ_{iso} (of ~0.6 ppm).

S4. DFT calculations on rehydrated Al MIL-53 and Ga MIL-53

Density functional theory (DFT) calculations were performed using the CASTEP code (version 16.11).[S3-S4] The PBE exchange correlation functional[S5] was adopted with dispersion corrections provided by the scheme of Tkatchenko and Scheffler.[S6] Calculations used a plane-wave cut-off of 50 Ry and the Brillouin zone was sampled on a Monkhorst-Pack grid[S7] with a k-point spacing of 0.04 2π Å^{-1}. Ultrasoft pseudopotentials were used with the inclusion of ZORA scalar relativistic effects. NMR calculations were performed on the all-electron wavefunction reconstructed through the GIPAW method.[S8-S10] Isotropic shieldings, σ_{iso}, were obtained from the trace of the absolute shielding tensor, $\boldsymbol{\sigma}$, and isotropic chemical shifts, δ_{iso}, given by $\delta_{iso} = -(\sigma_{iso} - \sigma_{ref})/m$,[S11] where $\sigma_{ref} = 271.31$ ppm and $m = -1.117$ were provided by comparison, *via* linear regression, of computed isotropic shieldings and experimental isotropic shifts of the hydroxyl and carboxylate ^{17}O species in calcined Al MIL-53. The quadrupolar coupling constant, $C_Q = eQV_{ZZ}/h$, and the asymmetry parameter, $\eta_Q = (V_{XX} - V_{YY})/V_{ZZ}$, are obtained directly from the principal components of the electric field gradient tensor, $\mathbf{V}$.[S11] Computed NMR data were extracted from CASTEP magres files using Python scripts based on the magres-format Python library.[S12]

Figure 3 of the main text show the presence of two different resonances in the carboxyl region of the ^{17}O NMR spectra of calcined, hydrated Al/Ga MIL-53. A previous experimental study by Ortiz *et al.*[S13] provided structural models of hydrated Al/Ga MIL-53 with two channels per unit cell (*cf.* the single channel seen in calcined Al/Ga MIL-53), where refinement of the powder X-ray diffraction (pXRD) data was based on information from ^{1}H solid-state NMR spectroscopy,[S13-S16] suggesting that two inequivalent water molecules are present in the material. The two different water molecules were proposed by Loiseau *et al.*[S14] and Lieder *et al.*[S15] to preferentially interact with the carboxylate oxygens through hydrogen bond donation, the latter suggesting a second ^{1}H resonance arising from a hydrogen bonding interaction between neighbouring water molecules. In

order to determine the relationship between carboxylate O inequivalence and the hydrogen-bonding network of encapsulated water molecules a computational investigation was undertaken, using the structural models (of hydrated Al and Ga MIL-53; Al-MIL-53_np_H_2O_dc and Ga-MIL-53_np_H_2O_dc respectively and models $\mathbf{A}_{Al}$ and $\mathbf{B}_{Ga}$ respectively herein) provided by Ortiz *et al.*[S13] as a starting point.

Models $\mathbf{A}_{Al}$ and $\mathbf{B}_{Ga}$ were treated separately, and subjected to geometry optimisation *via* periodic DFT calculations as described above. All atomic positions and the unit cell parameters were allowed to vary, giving $\mathbf{A}_{Al}'$ and $\mathbf{B}_{Ga}'$, respectively. The metal ions in $\mathbf{A}_{Al}'$ and $\mathbf{B}_{Ga}'$ were then "swapped" and the structures re-optimised to provide models with the hydrogen-bonding arrangement of model **B** with Al-centred metal nodes and **A** with Ga-centred metal nodes, termed $\mathbf{B}_{Al}'$ and $\mathbf{A}_{Ga}'$, respectively. Re-optimisation did not result in significant movement of water molecules. NMR parameters were then predicted for all models using GIPAW calculations, as described above.

Figure S4.1 shows the hydrogen-bonding arrangements found in the literature ($\mathbf{A}_{Al}$ and $\mathbf{B}_{Ga}$) and DFT-optimised ($\mathbf{A}_{Al}'$ and $\mathbf{B}_{Ga}'$) models, with the two crystallographically-distinct pores labelled I and II in each case. On optimisation, the cell volumes are found to decrease from 1888 Å^3 in $\mathbf{A}_{Al}$ to 1830 Å^3 in $\mathbf{A}_{Al}'$ and from 1928 Å^3 in $\mathbf{B}_{Ga}'$ to 1905 Å^3 in $\mathbf{B}_{Ga}'$, resulting in a narrowing of the pores. The most significant movement is seen in the water molecules, which, as a result, have shorter hydrogen-bonding contacts in the optimised structure than in those obtained from XRD. In A-type structures, both pores remain different in terms of their hydrogen-bonding arrangements. In pore I, water molecules donate two inequivalent hydrogen bonds to carboxylate oxygen centres and the water oxygen accepts a hydrogen bond from the metal hydroxyl. In pore II, the hydrogen bonds being donated from water to the carboxylate oxygen atoms are more alike, though with significantly different OH···O angles. For the B-type structure, proposed for hydrated Ga MIL-53, pores I and II exhibit differently orientated water molecules; in pore I the O–H bonds of the water molecules lie in near planarity with the crystallographic *ac* plane and,

therefore, do not donate hydrogen bonds to the carboxylate oxygens, whereas in pore II the waters lie perpendicular to the *ac* plane and so have shorter OH···O contacts with the carboxylate linkers. On optimisation, however, as can be seen in the schematic of $\mathbf{B}_{\mathrm{Ga}}'$ in Figure S4.1, the two formerly crystallographically-inequivalent pores become equivalent in terms of their sets of hydrogen-bonding interactions (though, due to retention of crystallographic symmetry, they do not become truly equivalent). These are also noted as having the same hydrogen-bonding arrangement as pore II in $\mathbf{A}_{\mathrm{Al}}'$.

The optimised structures, $\mathbf{A}_{\mathrm{Al}}'$ and $\mathbf{B}_{\mathrm{Ga}}'$, are significantly lower in energy (by 14.8 eV and 17.2 eV respectively) than the starting crystal structures, emphasising the stabilising effect of hydrogen bond formation between the carboxylate linkers and water molecules. Under close inspection of $\mathbf{A}_{\mathrm{Al}}'$ and $\mathbf{B}_{\mathrm{Ga}}'$, it is found that, for either of the two types of hydrogen-bonding arrangement found, the same set of carboxylate oxygen atoms accept hydrogen bonds from water. Omission of the water molecules (see Figure S4.2) reveals that the geometry of the narrow-pore framework exhibits a set of relatively short carboxylate to hydroxyl internuclear distances and a set of relatively large distances carboxylate to hydroxyl distances. It follows, therefore, that the former would be preferentially involved in hydrogen bonding with water.

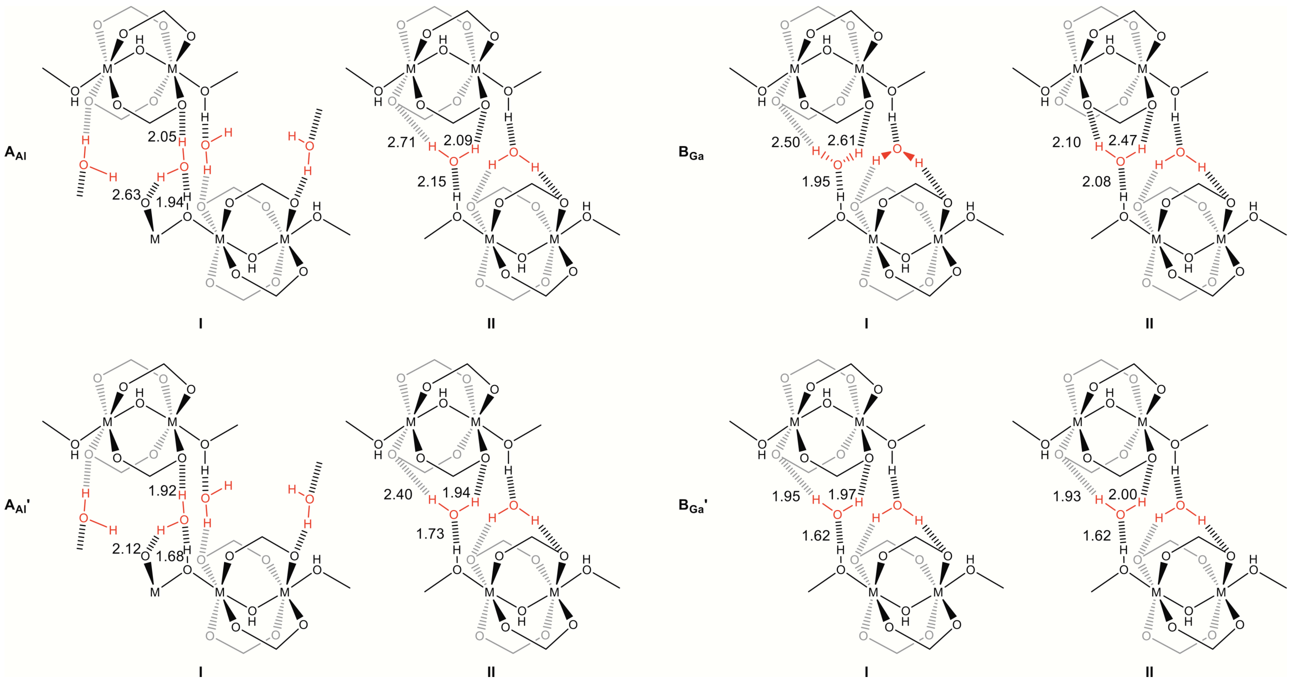

Figure S4.1 Schematic representations of the two chemically-inequivalent pores in the crystallographically-determined structures,[S13] $\mathbf{A}_{\mathbf{Al}}$ and $\mathbf{B}_{\mathbf{Ga}}$, and DFT-optimised structures, $\mathbf{A}_{\mathbf{Al}}'$ and $\mathbf{B}_{\mathbf{Ga}}'$, highlighting the hydrogen-bonding around encapsulated water molecules with selected distances in Å. The BDC aromatic rings are omitted for clarity.

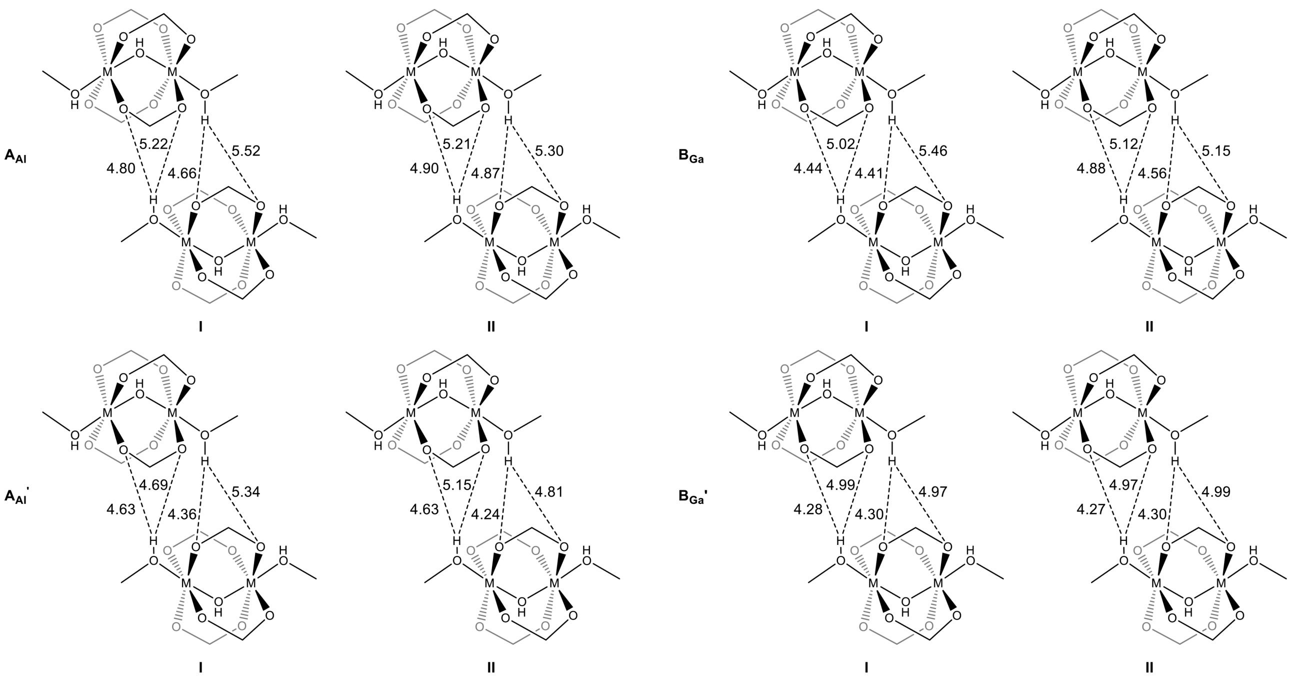

Figure S4.2 Schematic representations of the structures shown in Figure S4.1 with water omitted, showing the distances (in Å) between hydroxyl and carboxylate oxygens across the pore.

Computed ^{17}O NMR data are given in Table S4.1, with calcined Al MIL-53 used to provide the reference shielding (see above). (Note that for calculations can determine both the magnitude and sign of C_Q, while only the absolute magnitude is typically available experimentally). Carboxylate ^{17}O centres fall into two groups, which become better defined on geometry optimisation. This can be related to the strengthening of hydrogen-bonding interactions as a result of optimisation, which in turn separates the chemical shifts of hydrogen-bonded and non-hydrogen-bonded species, with predicted shifts of ~225 ppm and ~255 ppm, respectively. Notably, each of the different models adopted exhibit the same set of hydrogen-bonded O centres, suggesting any motion of encapsulated water would not exchange the carboxylate oxygens, in agreement with their experimental observation as inequivalent. Models **A** and **B** are very close in terms of their DFT energies, and so the predominance of either hydrogen-bonding arrangement or exchange between such structures is not ruled out by calculations. DFT-predicted quadrupolar parameters based on optimised geometries are within experimental error, with the exception of the hydroxyl oxygen in calcined Al MIL-53, where, as found previously,[S1] the magnitude of the C_Q is overestimated with respect to experiment.

Table S4.1 Computed ^{17}O NMR parameters for calcined Al MIL-53, $\mathbf{A_{Al}}$, $\mathbf{A_{Al}}'$, $\mathbf{B_{Al}}'$, $\mathbf{B_{Ga}}$, $\mathbf{B_{Ga}}'$ and $\mathbf{A_{Ga}}'$.

^{17}O species	δ_{iso} (ppm)	C_Q / MHz	η_Q	ΔE / eV
Calcined Al MIL-53				
carboxylate	230[a]	7.3	0.8	
hydroxyl	20[a]	–8.3	0.8	
$\mathbf{A_{Al}}$				14.8[b]
carboxylate 1	261	7.3	0.8	
carboxylate 2	269	7.8	0.5	
carboxylate 3	262	7.5	0.7	
carboxylate 4	280	7.6	0.5	
carboxylate 5	249	8.3	0.6	
carboxylate 6	214	8.3	0.7	
carboxylate 7	239	8.6	0.5	
carboxylate 8	203	8.0	0.8	
hydroxyl 1	8	6.1	1.0	
hydroxyl 2	1	–6.5	1.0	

water 1	1	10.5	0.7	
water 2	0	9.9	0.8	
**A_{Al}'**				0.0[b]
carboxylate 1	250	7.1	0.8	
carboxylate 2	255	7.4	0.7	
carboxylate 3	271	7.5	0.7	
carboxylate 4	274	7.7	0.5	
carboxylate 5	225	7.4	0.7	
carboxylate 6	230	7.9	0.8	
carboxylate 7	224	8.6	0.6	
carboxylate 8	226	8.8	0.6	
hydroxyl 1	24	6.4	0.9	
hydroxyl 2	23	6.4	1.0	
water 1	−5	9.1	0.8	
water 2	−9	9.0	0.8	
**B_{Al}'**				0.0[b]
carboxylate 1	267	7.4	0.6	
carboxylate 2	264	7.4	0.6	
carboxylate 3	264	7.4	0.6	
carboxylate 4	262	7.3	0.7	
carboxylate 5	225	8.4	0.7	
carboxylate 6	226	8.4	0.7	
carboxylate 7	226	8.4	0.7	
carboxylate 8	228	8.3	0.7	
hydroxyl 1	22	6.4	1.0	
hydroxyl 2	22	6.4	1.0	
water 1	−8	9.4	0.7	
water 2	−8	9.3	0.7	
**B_{Ga}**				17.2[c]
carboxylate 1	250	7.5	0.6	
carboxylate 2	267	6.5	0.8	
carboxylate 3	263	−6.7	1.0	
carboxylate 4	289	7.0	0.7	
carboxylate 5	253	8.3	0.7	
carboxylate 6	250	8.9	0.5	
carboxylate 7	250	8.3	0.6	
carboxylate 8	228	8.7	0.6	
hydroxyl 1	34	8.0	0.7	
hydroxyl 2	19	8.0	0.6	
water 1	33	11.6	0.6	
water 2	3	10.5	0.8	
**B_{Ga}'**				0.0[c]
carboxylate 1	278	6.9	0.7	

carboxylate 2	277	6.8	0.7	
carboxylate 3	277	6.8	0.7	
carboxylate 4	275	6.8	0.8	
carboxylate 5	230	8.6	0.6	
carboxylate 6	231	8.6	0.6	
carboxylate 7	231	8.6	0.6	
carboxylate 8	232	8.5	0.6	
hydroxyl 1	46	8.1	0.3	
hydroxyl 2	46	8.1	0.3	
water 1	−5	9.4	0.7	
water 2	−4	9.3	0.7	
$\mathbf{A_{Ga}}'$				0.3[c]
carboxylate 1	261	6.5	0.9	
carboxylate 2	264	6.9	0.8	
carboxylate 3	285	7.0	0.7	
carboxylate 4	286	7.4	0.6	
carboxylate 5	239	7.8	0.8	
carboxylate 6	232	7.9	0.8	
carboxylate 7	226	8.9	0.6	
carboxylate 8	230	9.1	0.5	
hydroxyl 1	48	7.9	0.3	
hydroxyl 2	48	8.1	0.4	
water 1	0	8.9	0.8	
water 2	−5	8.9	0.8	

[a] used for reference shielding [b] relative energy *vs* $\mathbf{A_{Al}}'$ [c] relative energy *vs* $\mathbf{B_{Ga}}'$

S5. NMR spectra of Al,Ga MIL-53

Figure S5.1 shows ^{17}O DOR spectra for the end member and mixed-metal MOFs acquired at 20.0 T. In each case, spectra are acquired with variable spinning rates to identify the isotropic resonances.

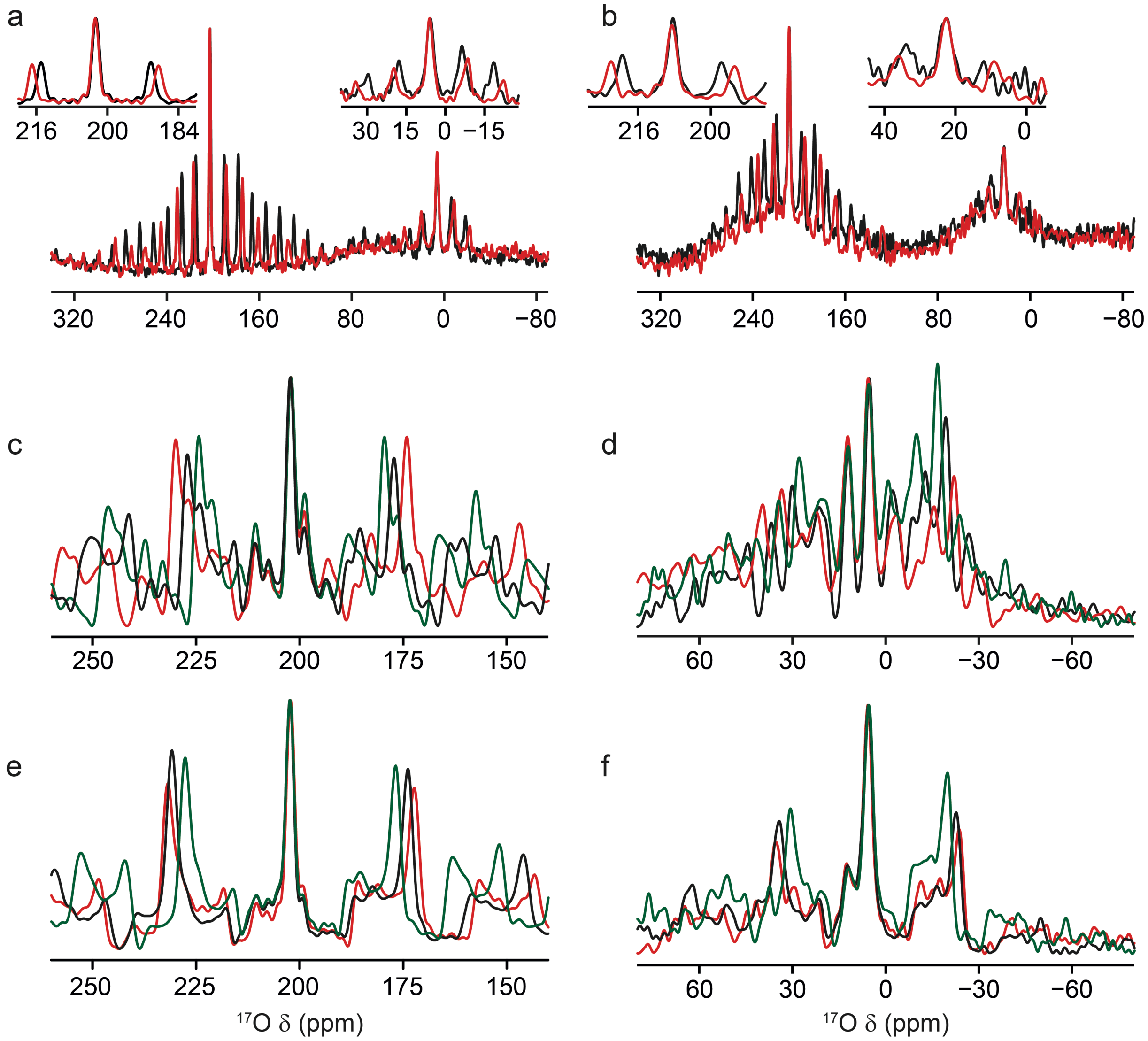

Figure S5.1 ^{17}O (20.0 T) DOR spectra of (a) Al and (b) Ga MIL-53 (and expansions of carboxyl and hydroxyl regions), (c, d) Al,Ga (50:50) MIL-53 and (e, f) Al,Ga (80:20) MIL-53. Rotation rates were (a) 1.4/6.9 kHz (black) or 1.6/7.7 kHz (red), (b) 1.25/6.3 kHz (black) or 1.55/7.9 kHz (red), (c, d) 1.48/7.4 kHz (black), 1.63/7.9 kHz (red) or 1.3/6.8 kHz (green), and (e, f) 1.65/8.0 kHz (black), 1.76/8.3 kHz (red) or 1.5/7.5 kHz (green).

^{27}Al MAS and MQMAS NMR spectra of calcined samples Al,Ga (80:20) MIL-53 and Al,Ga (50:50) MIL-53 are shown in Figure S5.2. Unlike the end member (see Figure S4.4), the spectra of the two mixed materials exhibit a distribution of NMR parameters (and therefore of local environments), confirming the presence of disorder arising from cation substitution.

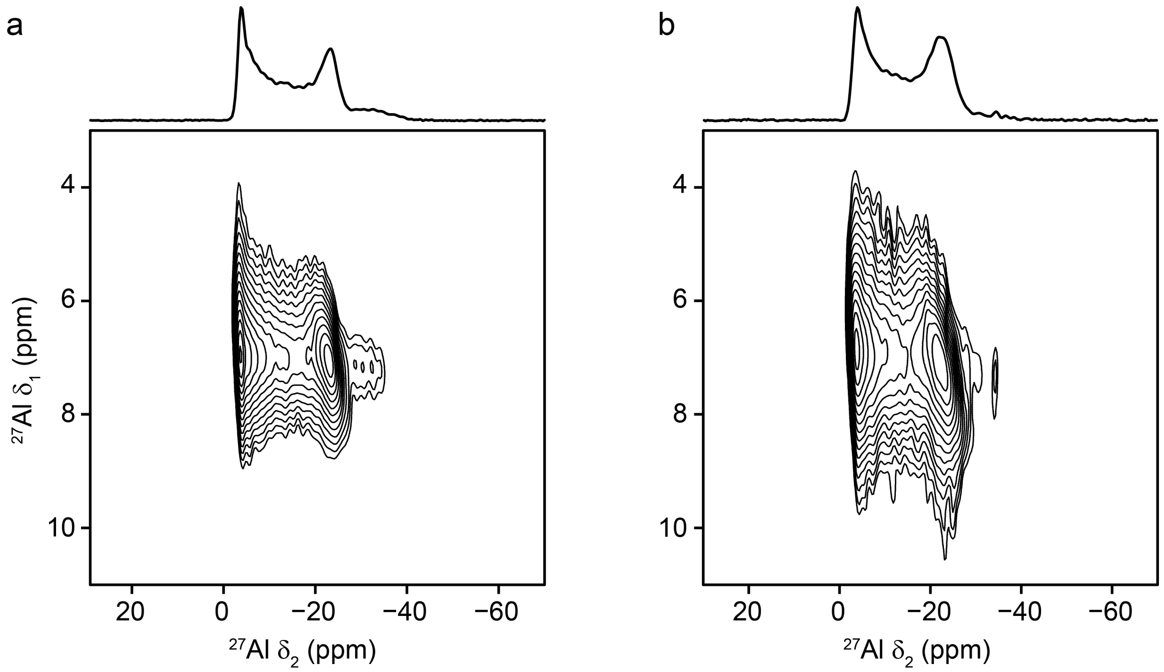

Figure S5.2 Al (14.1 T, 16 kHz) MQMAS NMR spectra of (a) Al,Ga (80:20) MIL-53 and (b) Al,Ga (50:50) MIL-53.

Insight into the magnitude of the distributions observed can be obtained by fitting a series of cross sections extracted parallel to δ_2. These confirm very little variation in δ_{iso}, which remains between 2.3 and 3 ppm, but a more significant distribution of C_Q, from 8.1 to 8.6 MHz for calcined Al,Ga (80:20) MIL-53 and 7.8 to 8.7 MHz for calcined Al,Ga (50:50) MIL-53. In contrast, for calcined Al MIL-53 (Figure S4.4) a single resonance is observed (with $C_Q = 8.4$ MHz, $\eta_Q = 0.04$ and $\delta_{iso} = 2.9$ ppm), reflecting the more ordered framework.

Figure S5.3 compares ^{17}O MAS and MQMAS NMR spectra of the hydrated and dehydrated forms of Al,Ga (50:50) MIL-53. Although closed pores are present (to some extent at least) in both forms, there is a change of the carboxyl resonance at higher shift,

indicating that these pores are not identical in the two forms and confirming that the closed pores do not result from a small amount of remaining water. (The carboxyl oxygen signal from the open pores in the dehydrated sample is overlapped with the second carboxyl resonance from the closed pores).

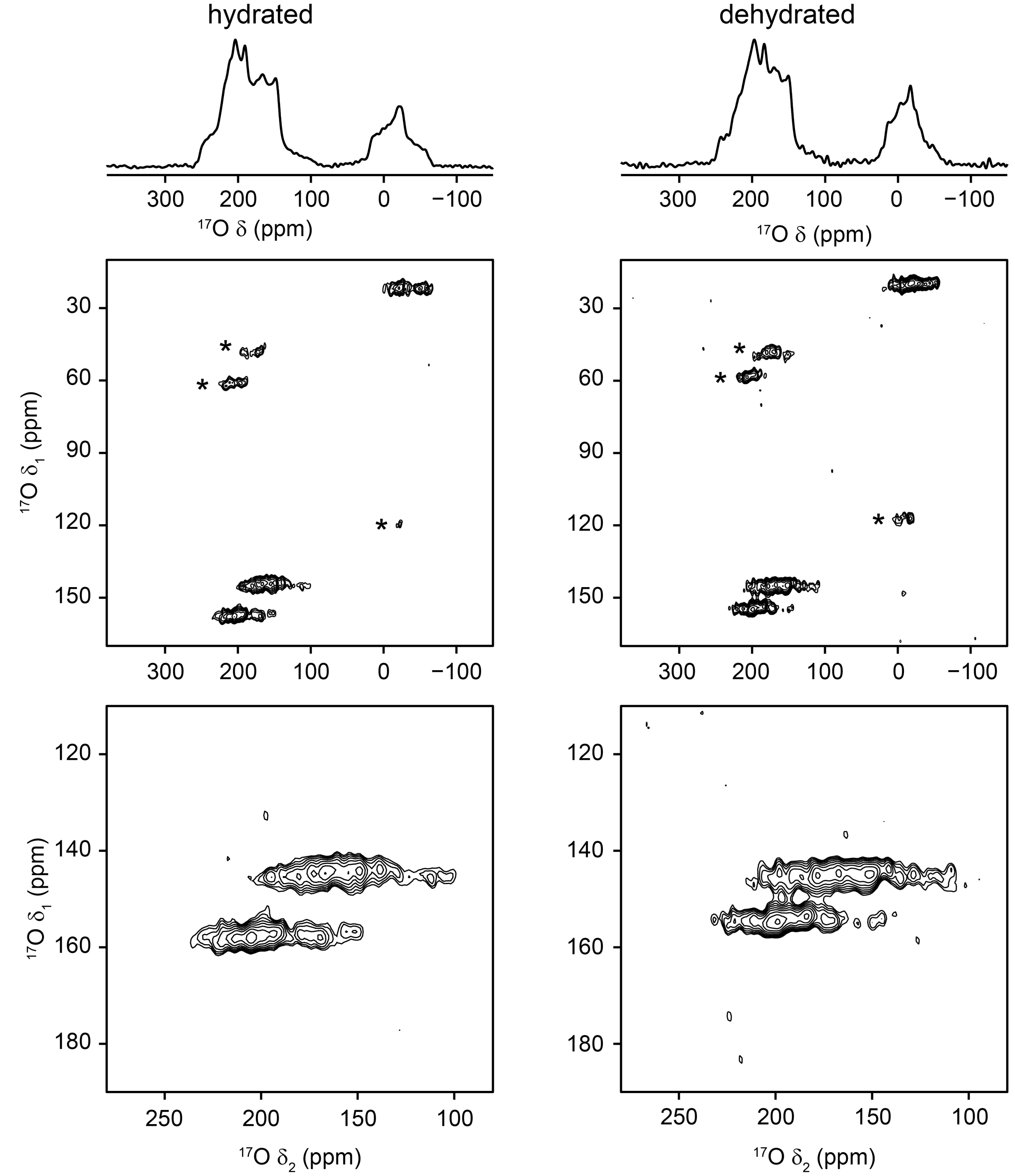

Figure S5.3 ^{17}O (14.1 T, 20 kHz) MAS and MQMAS NMR spectra (with expansion of the carboxyl region) of hydrated and dehydrated Al,Ga (50:50) MIL-53.

Differences in the exact nature of the closed pores observed in the hydrated and dehydrated forms can also be seen in the ^{13}C CP MAS spectra of Al,Ga (50:50) MIL-53 and Al,Ga (80:20) MIL-53 shown in Figure S5.4, with different chemical shifts seen for the carboxyl carbons in each form.

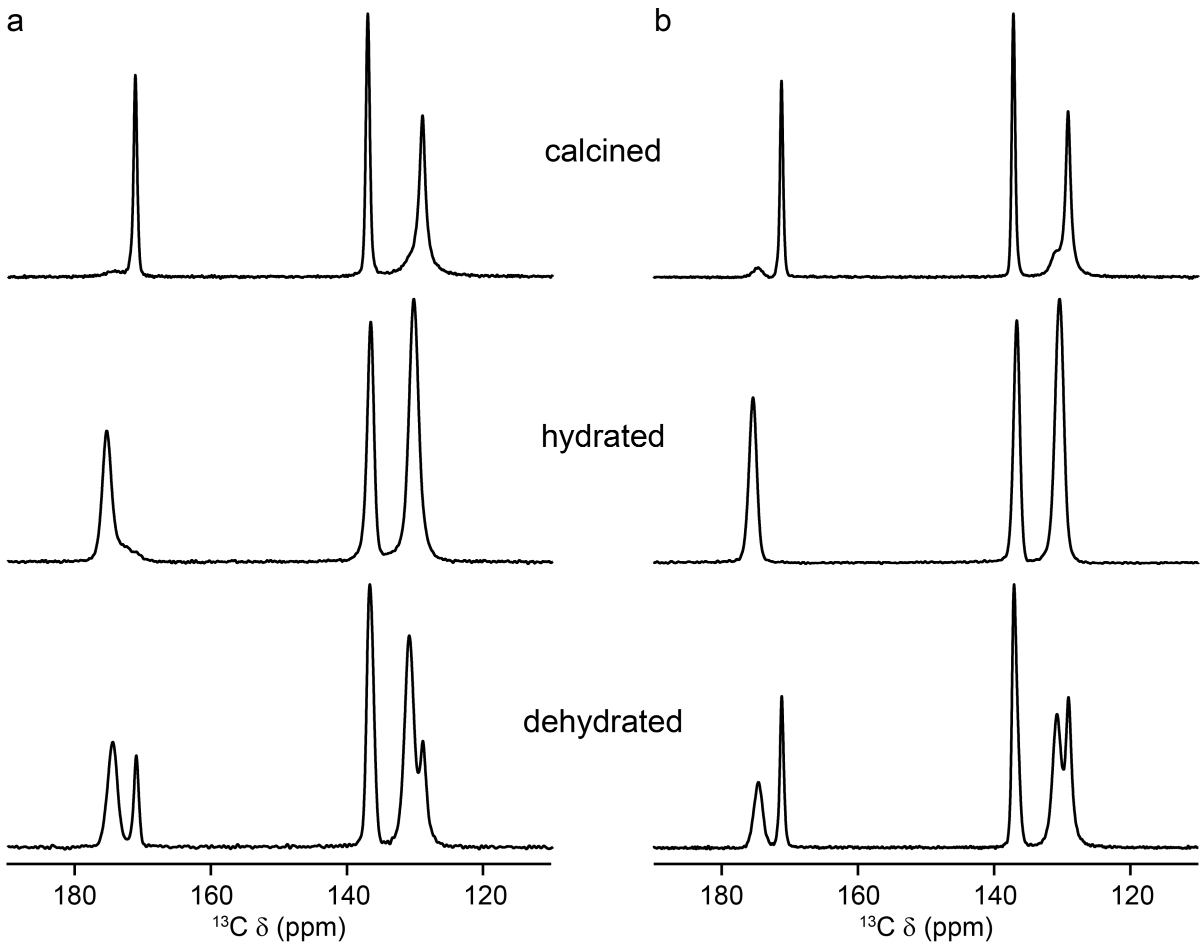

Figure S5.4 ^{13}C (14.1 T, 12.5 kHz) CP MAS NMR spectra of calcined, hydrated and dehydrated (a) Al,Ga (50:50) MIL-53 and (b) Al,Ga (80:20) MIL-53

S6. Powder XRD, EDX and SEM measurements

Figures S6.1 and S6.2 show powder XRD patterns of as-made, calcined, rehydrated and dehydrated Al MIL-53 and Ga MIL-53, respectively. For Al MIL-53, as-made form converts to a large-pore form upon calcination, which can then be reversibly interconverted in the narrow-pore hydrated form. However, for Ga MIL-53, the final step is not as reversible, with the closed-pore form still observed upon dehydration. Figure S6.2 also reveals the partial decomposition of Ga MIL-53, seen previously in the ^{17}O MAS NMR spectra.

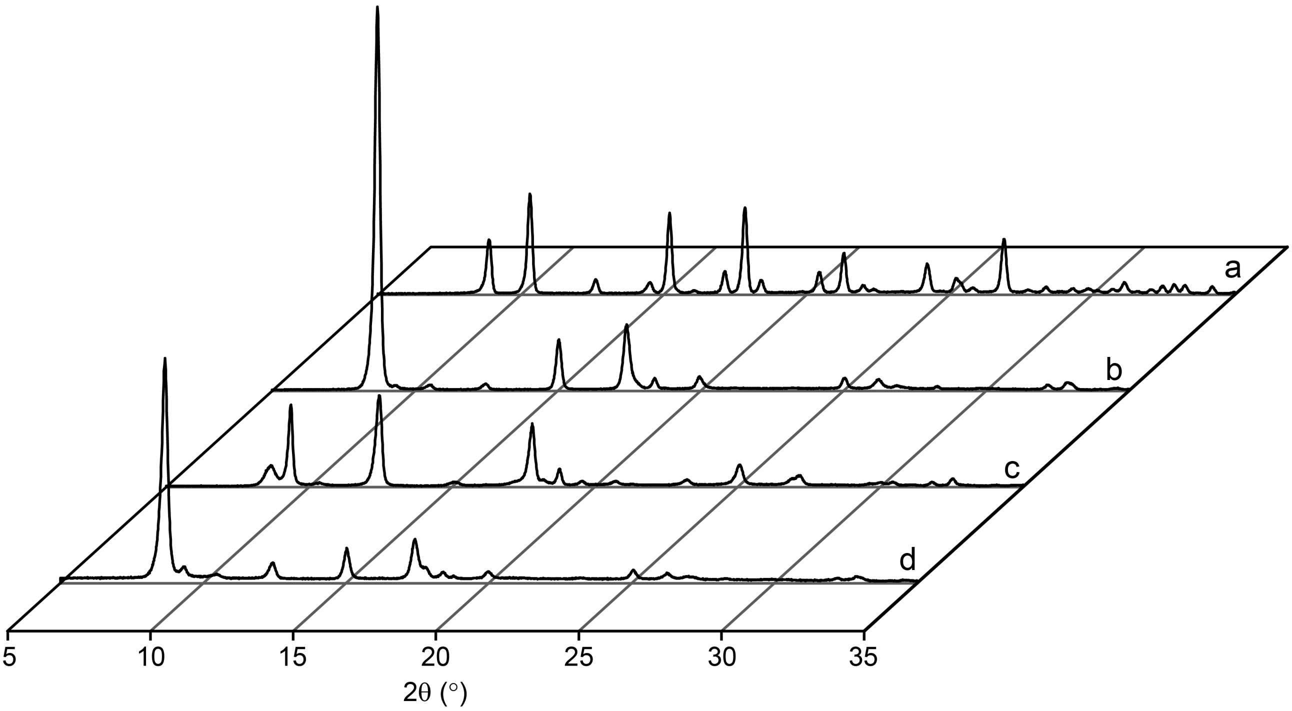

Figure S6.1 Powder XRD patterns of Al MIL-53 in its (a) as-made, (b) calcined open-pore, (c) hydrated closed-pore and (d) dehydrated open-pore forms.

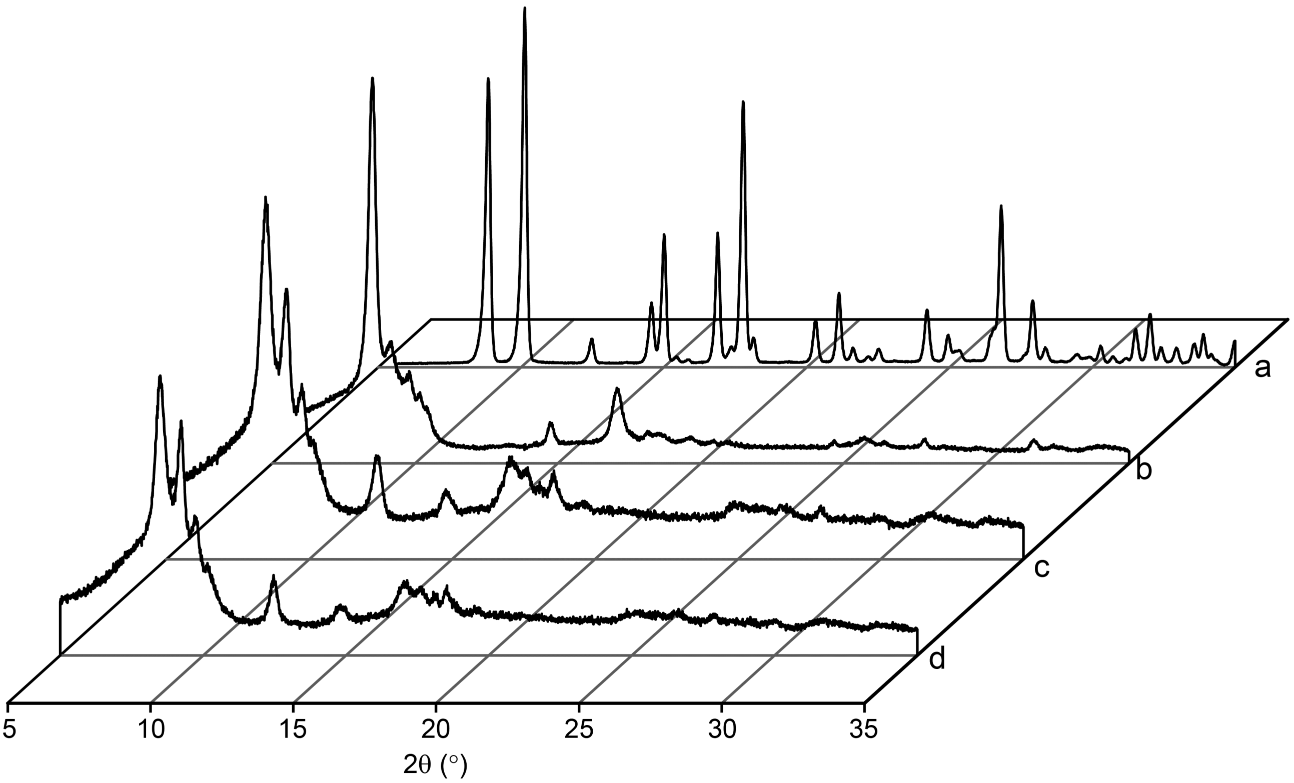

Figure S6.2 Powder XRD patterns of Ga MIL-53 in its (a) as-made, (b) calcined open-pore, (c) hydrated closed-pore and (d) dehydrated closed-pore forms.

Figures S6.3 and S6.4 show the Rietveld fits of the powder XRD pattern of as-made Al,Ga MIL-53 with 50:50 and 80:20 stoichiometric ratios, respectively. The unit cell parameters derived from these fits are given in Tables S6.1 and S6.2.

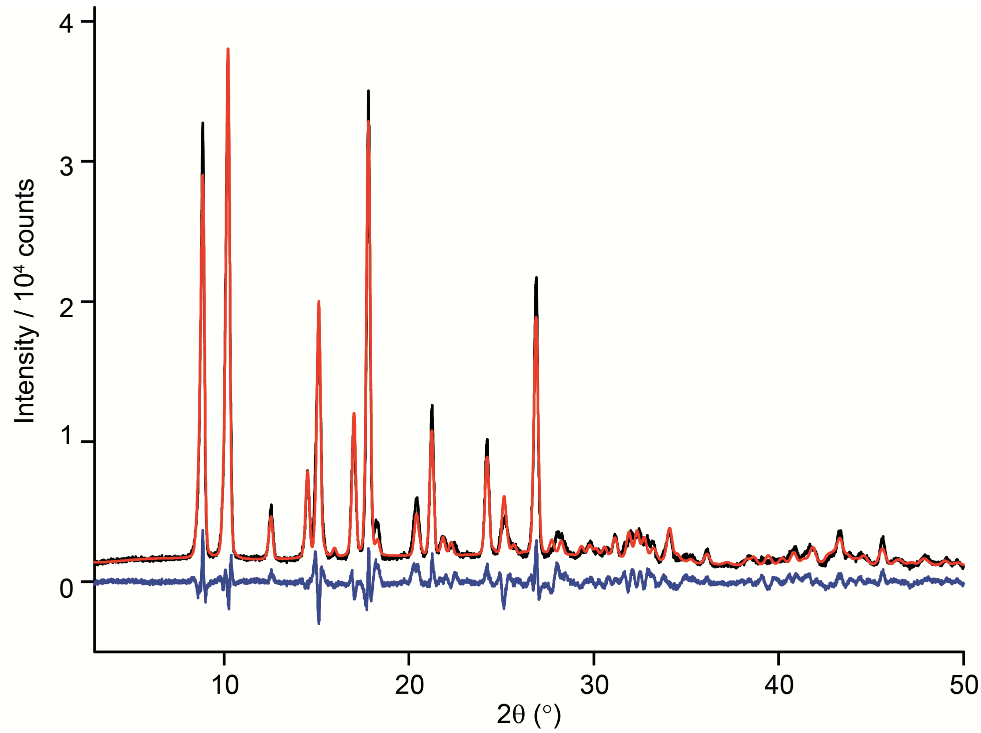

Figure S6.3 Rietveld fit of the powder XRD pattern for as-made Al,Ga (50:50) MIL-53.

Table S6.1 Unit cell parameters derived from the powder XRD pattern in Figure S6.3, for as-made Al,Ga (50:50) MIL-53.

Parameter	
R_{wp} (%)	11.03
R_{exp} (%)	1.84
space group	*Pnma*
a / Å	17.220 (1)
b / Å	6.6553 (7)
c / Å	12.1545 (7)
volume / Å^3	1393.5 (2)

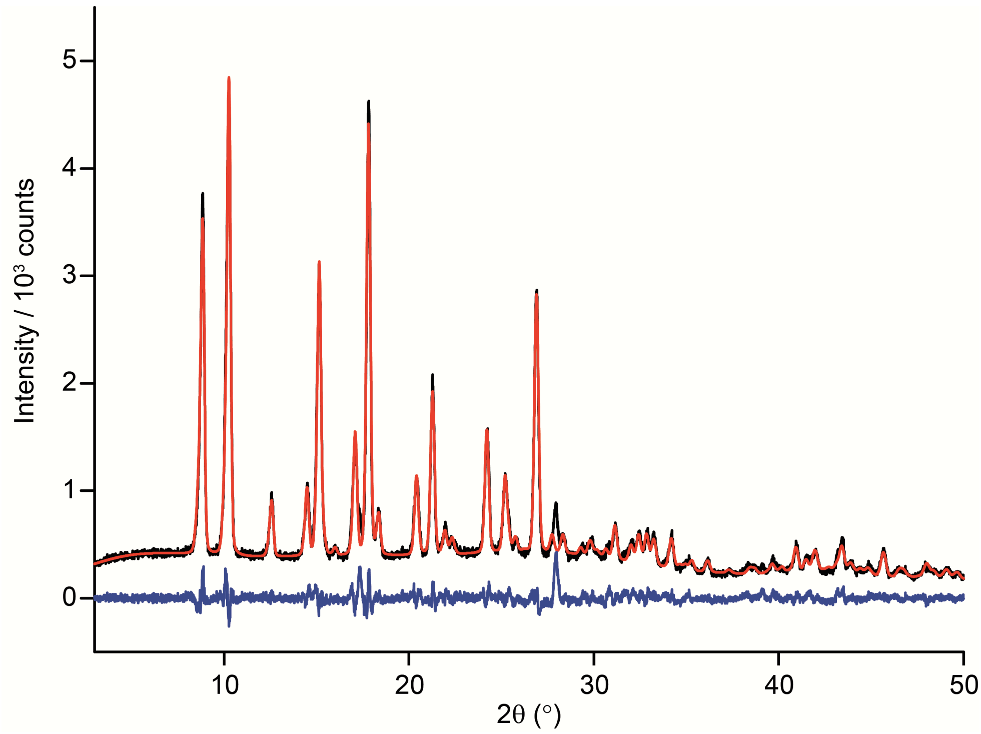

Figure S6.4 Rietveld fit of the powder XRD pattern for as-made Al,Ga (80:20) MIL-53.

Table S6.2 Unit cell parameters derived from the powder XRD pattern in Figure S6.4, for as-made Al,Ga (80:20) MIL-53.

Parameter	
R_{wp} (%)	7.68
R_{exp} (%)	4.33
space group	*Pnma*
a / Å	17.118 (1)
b / Å	6.6284 (6)
c / Å	12.1666 (6)
volume / Å^3	1380.5 (2)

A SEM image of the sieved powder for a sample of calcined Al,Ga MIL-53 with a nominal starting composition of 50:50 is shown in Figure S6.5. Table S6.3 and Figure S6.6 show the individual (and average) EDX measurements of Al and Ga content (%) for samples of calcined Al,Ga MIL-53 with nominal starting compositions of 50:50 and 80:20. For the

50:50 starting composition, the average value for the two samples studied are 72:28 and 74:26, respectively, in good agreement with the values determined using NMR spectroscopy. For the 80:20 sample, an average composition of 84:16 is found.

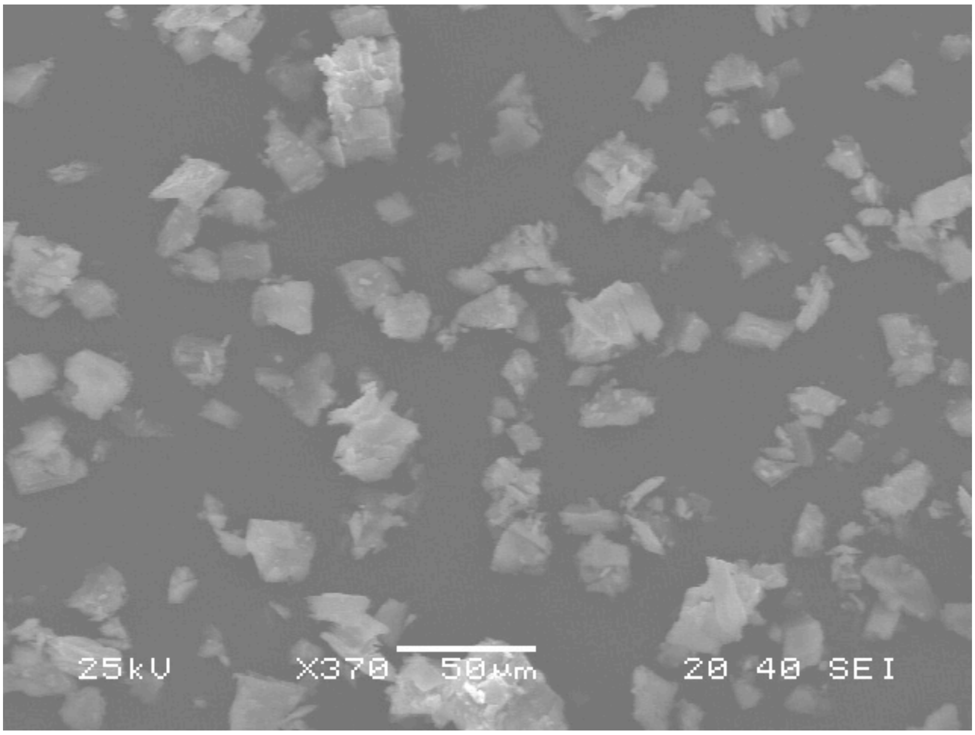

Figure S6.5 SEM image of a sieved powdered sample of calcined Al,Ga (50:50) MIL-53.

Table S6.3 EDX measurements of Al and Ga content (%) for samples of calcined Al,Ga MIL-53 with nominal starting compositions of 50:50 and 80:20.

Starting composition 50:50				Starting composition 80:20	
Sample 1		Sample 2		Sample 1	
Al (%)	Ga (%)	Al (%)	Ga (%)	Al (%)	Ga (%)
58.50	41.50	71.37	28.63	73.85	26.15
61.17	38.83	68.14	31.86	71.66	28.34
72.77	27.23	61.52	38.48	75.85	24.16
86.37	13.63	74.63	25.37	60.57	39.43
68.74	31.26	64.70	35.30	90.47	9.53
69.29	30.71	85.11	14.89	90.74	9.26
71.71	28.29	70.93	29.07	65.96	34.04
62.81	37.19	84.35	15.65	89.27	10.73
68.98	31.02	71.78	28.22	91.95	8.05
69.03	30.97	80.24	19.76	91.29	8.71
72.68	27.32	84.30	15.70	92.95	7.05
67.87	32.13	84.14	15.86	90.31	9.69
74.53	25.47	71.51	28.49	94.31	5.69
87.48	12.52	74.56	25.44	91.24	8.76
81.04	18.96	65.01	34.99	90.77	9.23
70.90	29.10	41.64	58.36	66.81	33.19
67.94	32.06	83.32	16.68	89.70	10.30
90.40	9.60	73.89	26.11	92.01	7.99
		80.73	19.27	91.80	8.20
		84.57	15.43	87.47	12.53
		79.35	20.65	86.26	13.74
		74.09	25.91	69.52	30.48
				89.18	10.82
72 ± 9	28 ± 9	74 ± 10	26 ± 10	84 ± 11	16 ± 11

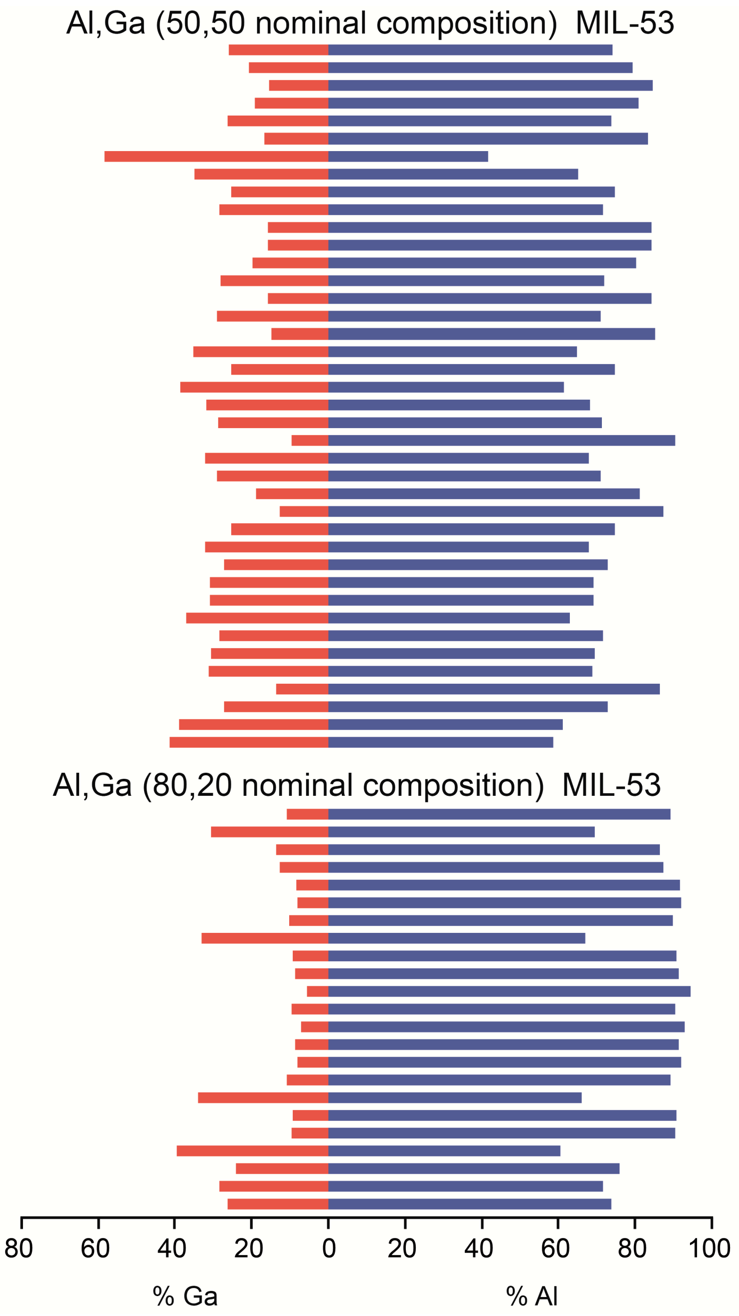

Figure S6.6 Al and Ga content (%) from EDX measurements for calcined Al,Ga MIL-53 with nominal starting compositions of 50:50 and 80:20.

Table S6.4 gives the results of Pawley fits of powder XRD patterns, carried out for as-made, calcined (large-pore, lp), hydrated (narrow-pore, np) and dehydrated Al,Ga MIL-53 with a nominal starting composition of 50:50. In all cases, the unit cell size is larger than that observed for Al MIL-53, confirming substitution of Ga. The corresponding powder XRD patterns are shown in the main text. Although, in principle, the presence of a np

phase in the calcined material could be the result of a small amount of hydration (as opposed to the presence of Ga in the material), dehydration was carried out *in-situ* to avoid any air exposure of the solid packed in the capillary.

Table S6.4 Results from Pawley fits of powder XRD patterns, carried out for as-made, calcined (large-pore, lp), hydrated (narrow-pore, np) and dehydrated Al,Ga (50:50) MIL-53.

	as-made	calcined		hydrated	dehydrated	
R_{wp} (%)	5.96	5.45		8.76	5.56	
R_{exp} (%)	1.82	3.16		2.61	1.79	
phase	as-made	lp	np	np (	lp	np
space group	*Pnma*	*Imma*	*Cc*	*Cc*	*Imma*	*Cc*
a / Å	17.220 (1)	6.641 (1)	19.592 (9)	19.608 (3)	6.648 (1)	19.53 (3)
b / Å	6.6570 (5)	16.774 (5)	7.576 (2)	7.6637 (8)	16.803 (6)	7.1136 (9)
c / Å	12.1546 (6)	12.751 (5)	6.583 (3)	6.6203 (9)	12.799 (2)	6.98 (1)
β (°)	-	-	105.09 (8)	104.17 (2)	-	101.5 (5)
volume / $Å^3$	1393.3 (2)	1420.4 (7)	943.4 (9)	964.6 (2)	1429.8 (7)	951 (3)

S7. Characterisation of $Sc_2(BDC)_3$

Attempts to synthesise Sc MIL-53 using DGC produced $Sc_2(BDC)_3$: a different, denser terephthalate MOF. The powder XRD pattern (Figure S7.1) confirms the formation of this material. The ^{13}C CP MAS NMR spectrum shown in Figure S7.2a confirms the presence of two distinct terephthalate linkers (in a 2:1 ratio), in agreement with previous work.[S16] The ^{1}H MAS spectrum in Figure S7.2b shows a single signal at ~7 ppm (corresponding to the aromatic H of the linker), and confirms the absence of hydroxyl groups. A single ^{45}Sc species is seen in Figure S7.2c, in agreement with previous work.

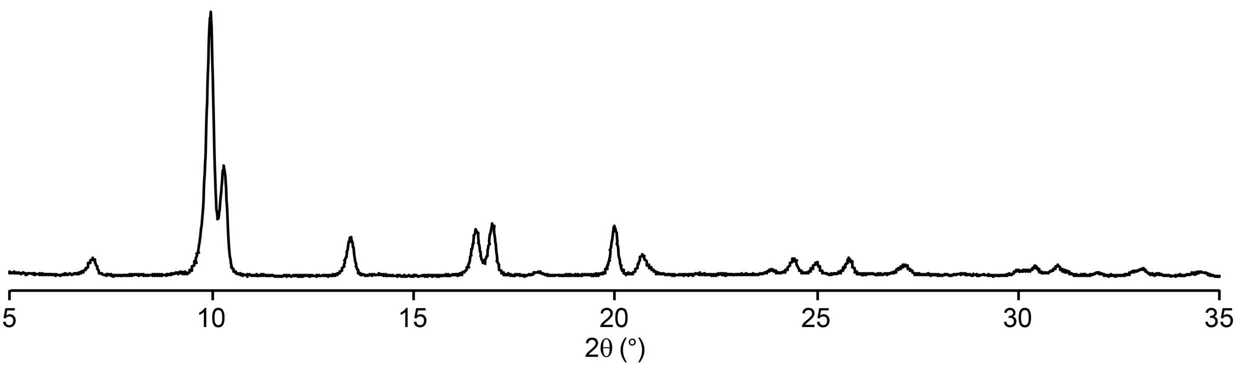

Figure S7.1 Powder XRD pattern of calcined $Sc_2(BDC)_3$.

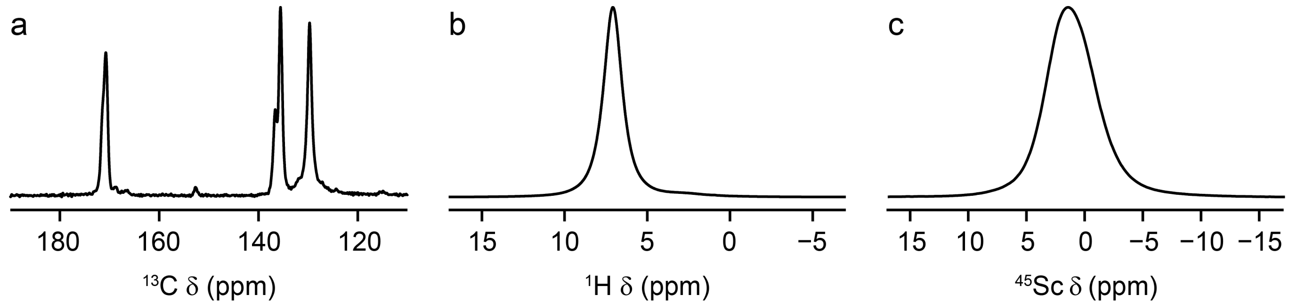

Figure S7.2 (a) ^{13}C (14.1 T, 12.5 kHz) CP MAS NMR, (b) ^{1}H (14.1 T, 12.5 kHz) spin echo and (c) ^{45}Sc (14.1 T, 20.0 kHz) MAS NMR spectra of calcined $Sc_2(BDC)_3$.

S8. Characterisation of mixed-phase Al MIL-53 and $Sc_2(BDC)_3$

DGC synthesis of a mixed-metal (Sc, Al) MIL-53 resulted in a two-phase mixture of $Sc_2(BDC)_3$ and Al MIL-53, as confirmed by powder XRD and solid-state NMR spectroscopy. The powder XRD pattern (Figure S8.1) shows peaks resulting from both the "BDC" phase and a MIL-53 phase, at positions very similar to those found for the two pure phase materials, suggesting limited, if any, cation substitution. This is supported also by the ^{13}C CP MAS spectrum (Figure S8.2a), which shows clear resolution of two ^{13}C resonances at 135.6 and 137.2 ppm, corresponding to the quaternary aromatic carbons in the two distinct structures. The ^{1}H MAS NMR spectrum (Figure S8.2b) confirms the presence of hydroxyl groups in the MIL-53 phase.

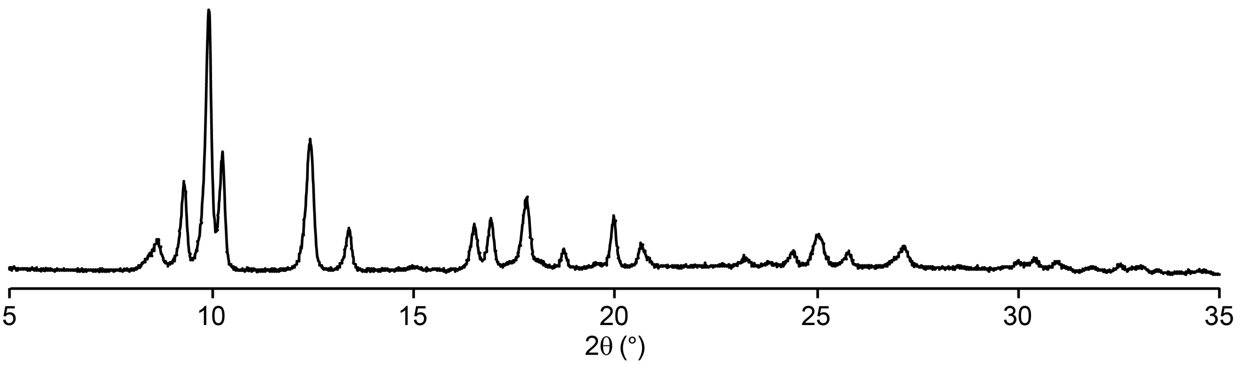

Figure S8.1 Powder XRD pattern of mixed-phase calcined $Sc_2(BDC)_3$ and Al MIL-53.

The ^{17}O MAS and MQMAS spectra of this sample (Figure S8.3) also confirm the presence of signals very similar to those found for the two separate phases. Upon hydration, the splitting of the carboxyl resonance from Al MIL-53 is observed (also shown in Figure S8.3).

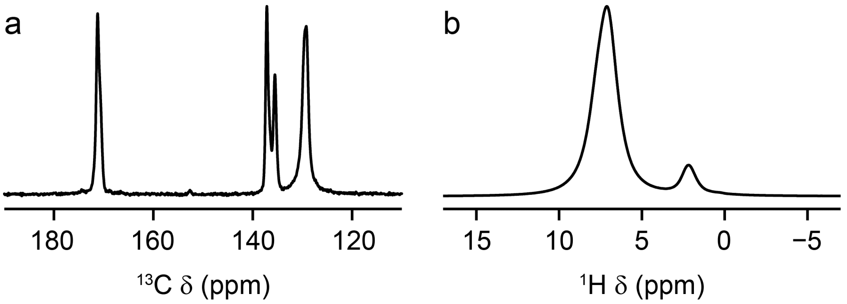

Figure S8.2 (a) ^{13}C (14.1 T, 12.5 kHz) CP MAS NMR and (b) ^{1}H (14.1 T, 12.5 kHz) spin echo MAS NMR spectra of mixed-phase calcined $Sc_2(BDC)_3$ and Al MIL-53.

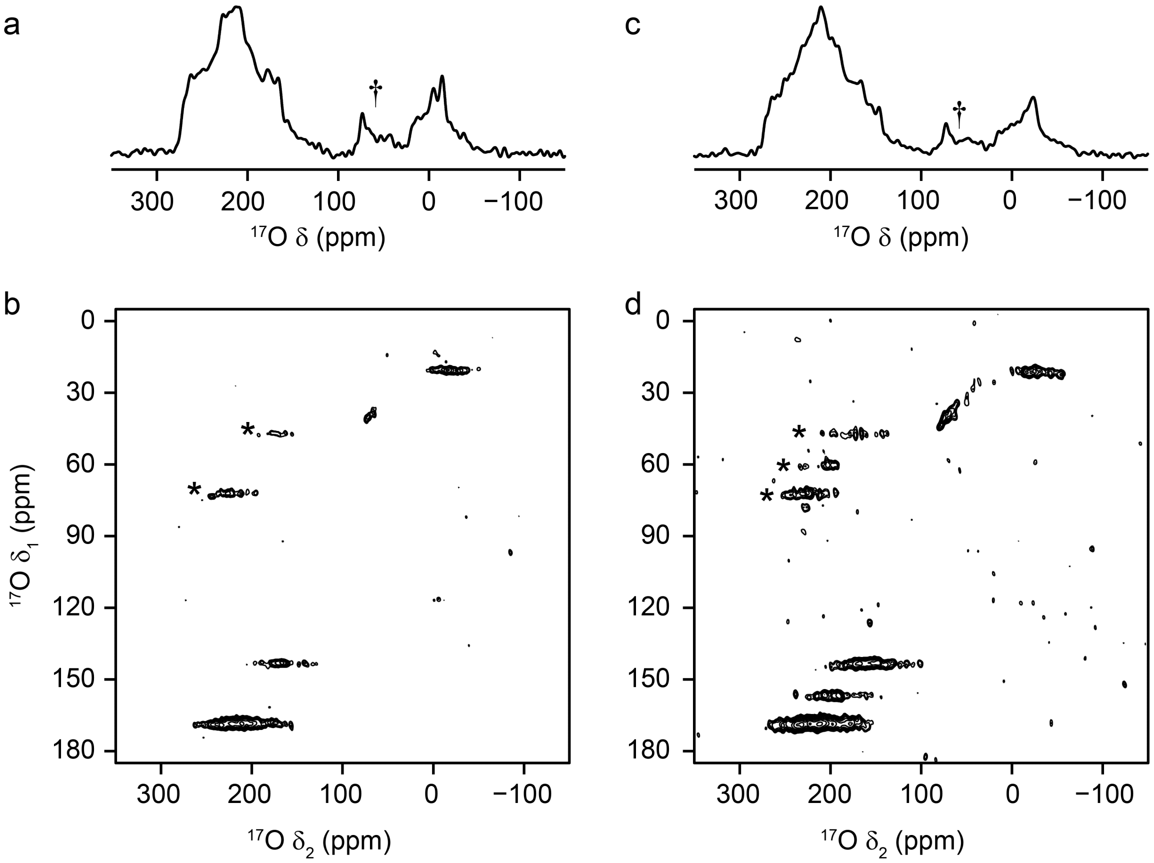

Figure S8.3 ^{17}O (14.1 T, 20 kHz) (a, c) spin echo MAS and (b, d) MQMAS NMR spectra of mixed-phase (a, b) calcined and (c, d) rehydrated $Sc_2(BDC)_3$ and Al MIL-53. Asterisks denote spinning sidebands.

S9. Additional ^{17}O experimental NMR parameters

Table S9.1 Additional experimental NMR parameters for the ^{17}O spectra shown in the main text and ESI. B_0 field strengths and MAS rates are given in the captions.

Figure	Experiment	Transients	Recycle interval / s	Increments	Increment / μs
Figures 2a-d	spin echo	4096	1		
Figure 2e	3QMAS	792	0.7	150	12.5
Figure 2f	3QMAS	960	0.75	154	10.0
Figures 3a-b	spin echo	4096	1		
Figure 3c	3QMAS	816	0.7	200	12.5
Figure 3d	3QMAS	1416	0.7	80	16.7
Figure 4a-d	spin echo	6114	1		
Figure 5a-d	3QMAS	480-576	0.75	100-120	12.5
Figure 6a	DOR	4552	1		
Figure 6b	DOR	5744	1		
Figure 6c	DOR	81920	1		
Figure 6d	DOR	57344	1		
Figure 7a	MAS	21504	2		
Figure 8a-d	spin echo	4096	1		
Figure 9a-b	spin echo	4096	1		
Figure 9c	3QMAS	720	0.7	150	12.5
Figure 9d	3QMAS	720	0.75	120	10.0
Figure S5.1a (black)	DOR	6584	1		
Figure S5.1b (black)	DOR	12120	1		
Figure S5.1c-d	DOR	73728	1		

(black)					
Figure S5.1c-d (green)	DOR	62552	1		
Figure S5.1e-f (red)	DOR	10312	1		
Figure S5.1e-f (green)	DOR	22544	1		
Figure S5.3(hyd)	MAS	4096	1		
Figure S5.3(dehyd)	MAS	4096	1		
Figure S5.3(hyd)	3QMAS	576	0.7	140	12.5
Figure S5.3(dehyd)	3QMAS	480	0.7	150	12.5
Figure S8.3a,c	spin echo	4096	1		
Figure S8.3b	3QMAS	816	0.7	180	12.5
Figure S8.3d	3QMAS	672	0.7	144	12.5

S10. References

S1. P. He, J. Xu, V. V. Terskikh, A. Sutrisno, H.-Y. Nie and Y. Huang, *J. Phys. Chem. C*, 2013, **117**, 16953–16960.

S2. J. P. S. Mowat, S. R. Miller, A. M. Z. Slawin, V. R. Seymour, S. E. Ashbrook and P. A. Wright, *Micropor. Mesopor. Mater.*, 2011, **142**, 322–333.

S3. S. J. Clark, M. D. Segall, C. J. Pickard, P. J. Hasnip, M. J. Probert, K. Refson, M. C. Payne, *Z. Kristallogr.*, 2005, **220**, 567−570.

S4. C. J. Pickard, F. Mauri, *Phys. Rev. B,* 2001, **63**, 245101.

S5. J. P. Perdew, K. Burke and M. Ernzerhof, *Phys. Rev. Lett.*, 1996, **77**, 3865–3868.

S6. A. Tkatchenko and M. Scheffler, *Phys. Rev. Lett.*, 2009, **102**, 073005 .

S7. H. J. Monkhorst and J. D. Pack, *Phys. Rev. B*, 1976, **13**, 5188–5192.

S8. C. Pickard and F. Mauri, in Calculation of NMR and EPR Parameters: Theory and Applications, ed. M. Kaupp, M. Buhl and V. G. Malkin, Wiley-VCH, Weinheim, 2004, pp. 265–278.

S9. C. J. Pickard and F. Mauri, *Phys. Rev. B*, 2001, **63**, 245101.

S10. J. R. Yates, C. J. Pickard and F. Mauri, *Phys. Rev. B*, 2007, **76**, 024401.

S11. S. E. Ashbrook and D. McKay, *Chem. Commun.*, 2016, **52**, 7186–7204.

S12. S. Sturniolo, T. F. G. Green, R. M. Hanson, M. Zilka, K. Refson, P. Hodgkinson, S. P. Brown and J. R. Yates, *Solid State Nucl. Magn. Reson.*, 2016, **78**, 64–70.

S13. G. Ortiz, G. Chaplais, J.-L. Paillaud, H. Nouali, J. Patarin, J. Raya and C. Marichal, *J. Phys. Chem. C*, 2014, **118**, 22021–22029.

S14. T. Loiseau, C. Serre, C. Huguenard, G. Fink, F. Taulelle, M. Henry, T. Bataille and G. Férey, *Chem. Eur. J.*, 2004, **10**, 1373–1382.

S15. C. Lieder, S. Opelt, M. Dyballa, H. Henning, E. Klemm and M. Hunger, *J. Phys. Chem. C*, 2010, **114**, 16596–16602.

S16. J. P. S. Mowat, S. R. Miller, J. M. Griffin, V. R. Seymour, S. E. Ashbrook, S. P. Thompson, D. Fairen-Jimenez, A.-M. Banu, T. Düren and P. A. Wright, *Inorg. Chem.*, 2011, **50**, 10844–10858.
